# Epigallocatechin-3-gallate alleviates bladder overactivity in a rat model with metabolic syndrome and ovarian hormone deficiency through mitochondria apoptosis pathways

**DOI:** 10.1038/s41598-018-23800-w

**Published:** 2018-03-29

**Authors:** Yi-Lun Lee, Kun-Ling Lin, Bin-Nan Wu, Shu-Mien Chuang, Wen-Jeng Wu, Yung-Chin Lee, Wan-Ting Ho, Yung-Shun Juan

**Affiliations:** 1 Graduate Institute of Medicine, College of Medicine, Kaohsiung Medical University, Kaohsiung, Taiwan; 2 Department of Urology, Sinying Hospital, Ministry of Health and Welfare, Tainan, Taiwan; 3 Graduate Institute of Clinical Medicine, College of Medicine, Kaohsiung Medical University, Kaohsiung, Taiwan; 4 Department of Obstetrics and Gynecology, Kaohsiung Medical University Hospital, Kaohsiung, Taiwan; 5 Department of Pharmacology, College of Medicine, Kaohsiung Medical University, Kaohsiung, Taiwan; 6 Translational Research Center, Cancer Center, Department of Medical Research, Kaohsiung Medical University, Kaohsiung, Taiwan; 7 Department of Urology, College of Medicine, Kaohsiung Medical University, Kaohsiung, Taiwan; 8 Department of Urology, Kaohsiung Medical University Hospital, Kaohsiung, Taiwan; 9 Department of Urology, Kaohsiung Municipal Ta-Tung Hospital, Kaohsiung, Taiwan; 10 Department of Urology, Kaohsiung Municipal Hsiao-Kang Hospital, Kaohsiung, Taiwan

## Abstract

Metabolic syndrome (MetS) and ovarian hormone deficiency could affect bladder storage dysfunction. Epigallocatechin-3-gallate (EGCG), a polyphenolic compound in green tea, has been shown to protect against ovarian hormone deficiency induced overactive bladder (OAB). The present study investigated oxidative stress induced by MetS and bilateral ovariectomy (OVX), and elucidated the mechanism underlying the protective effect of EGCG (10 umol/kg/day) on bladder overactivity. Rats were fed with high fat high sugar (HFHS) diet to induce MetS and received ovariectomy surgery to deprive ovarian hormone. By dieting with HFHS for 6 months, rats developed MetS and OAB. MetS + OVX deteriorated bladder storage dysfunction more profound than MetS alone. MetS and MetS + OVX rats showed over-expression of inflammatory and fibrosis markers (1.7~3.8-fold of control). EGCG pretreatment alleviated storage dysfunction, and protected the bladders from MetS and OVX - induced interstitial fibrosis changes. Moreover, OVX exacerbated MetS related bladder apoptosis (2.3~4.5-fold of control; 1.8~2.6-fold of Mets group), enhances oxidative stress markers (3.6~4.3-fold of control; 1.8~2.2-fold of Mets group) and mitochondrial enzyme complexes subunits (1.8~3.7-fold of control; 1.5~3.4-fold of Mets group). EGCG pretreatment alleviated bladder apoptosis, attenuated oxidative stress, and reduced the mitochondrial and endoplasmic reticulum apoptotic signals. In conclusions, HFHS feeding and ovarian hormone deficiency enhances the generation of oxidative stress mediated through mitochondrial pathway. EGCG reduced the generation of oxidative stress and lessened bladder overactivity.

## Introduction

Estrogen is a unique hormone that modulates various physiological conditions in females including voiding function^1^. Ovarian hormone deficiency is associated with the increased risk of the metabolic syndrome (MetS) in the female^2^. People with MetS have two to three-fold risk of attributing to overactive bladder (OAB) in adults^3,4^. Ovarian hormone deficiency in the ovariectomy (OVX) rabbits appeared bladder hyperactivities^5^. Experimentally, OVX was applied to mimic the physiological conditions of ovarian hormone deficiency and to induce bladder overactivity in a rat model.

Our previous investigation demonstrated that ovarian hormone deficiency in the OVX rats resulted in diminishing bladder compliance, increasing oxidative damage and interstitial fibrosis, and enhancing bladder mucosa apotosis^6^. Both ovarian hormone deficiency and MetS have been associated with the bladder overactivity and lower urinary tract symptoms (LUTS)^7^. The relation between MetS and ovarian hormone deficiency has become an important issue because of a high prevalence of such combined status in aging women.

Mitochondrial function is considered relating to MetS associated oxidative stress^8^, but the mechanisms of MetS and ovarian hormone deficiency in relation to bladder dysfunction are still not clearly elucidated. Mitochondria are the principal source of reactive oxygen species (ROS) in cells as a result of imperfectly coupled electron transport. Hyperglycemia could trigger superoxide overproduction by mitochondria^8^. Mitochondria may also induce apoptosis by altering cellular redox potential via increased ROS production^9,10^. In the fructose-fed animal model^11^, MetS with hyperlipidemia and hyperglycemia was found to result in ROS production and impair mitochondrial function to produce ATP.

Moreover, endoplasmic reticulum (ER) could lead to pro-apoptotic unfolded protein response, including the induction of CCAAT/enhancer-binding protein-homologous protein (CHOP), 78-KDa glucose-regulated protein (GRP 78) and caspase-12^12,13^. The apoptotic processes is induced by ER stress through CHOP and/or caspase-12 dependent pathways in diabetic kidney, which may contribute to the development of diabetic nephropathy^14^. Alterations in the functions of mitochondria and ER might contribute to the development of metabolic disorders^15^. Thus, decreasing oxidative stress as well as enhancing mitochondrial function might be important therapeutic strategies for MetS^8^.

Epigallocatechin-3-gallate (EGCG) extracted from green tea is well known as strong antioxidant compounds to exhibit anti-oxidative and anti-inflammatory properties^16^. The EGCG compound was found to induce apoptosis via the generation of reactive oxygen species (ROS), which led to the activation of p-38, caspase-3 and the cleavage of poly ADP-ribose polymerase (PARP)^17^. EGCG was also reported to enhance the activity of antioxidant enzymes, superoxide dismutase (Mn-SOD and Cu/Zn-SOD) and catalase^18^. In addition, EGCG was found to be a powerful hydrogen-donating antioxidant and a free radical scavenger of ROS^6,19^. EGCG showed the ability of alleviating oxidative damage and bladder hyperactivities in OVX rats^6^. Aging females are susceptible to OVX ovarian hormone deficiency combined with MetS.

In spite of the above progresses, the cellular mechanism of ovarian hormone deficiency and MetS in interfering bladder activity and voiding behavior is still not clear. In extension of our previous investigations of EGCG pretreatment for bladder overactivity and voiding dysfunction in female rats^5,6^, the present work was focused on elucidating the potential mechanisms underlying the oxidative stress and apoptosis mediated by mitochondria and ER pathways as well as the antioxidant effect of EGCG on MetS and ovarian hormone deficiency associated bladder overactivity in a rat model. To achieve these specific aims, we investigated the expression levels of oxidative stress markers, ER stress markers, and mitochondrial proteins associated with apoptosis and respiratory enzyme complexes, and evaluated the antioxidant effect of EGCG pretreatment on bladder overactivity.

## Materials and Methods

### Animals and feeding protocol

The experimental procedures were approved by the Committee for the Use of Experimental Animal of Kaohsiung Medical University and were adhered to the guidelines of the National Institute of Health for the use of the experimental animals. Total 52 female Sprague-Dawley rats purchased from Animal Center of BioLASCO (Taipei, Taiwan) initially weighing 200–250 g were divided into five different groups: (a) normal rat chow diet (the control) group, (b) MetS induced with the high fat and high sugar (HFHS) dieting (the MetS) group, (c) MetS induced with the HFHS dieting plus bilateral OVX (the MetS + OVX) group, (d) MetS induced with the HFHS dieting plus bilateral OVX combined with EGCG (10 umol/kg/day) intraperitoneal (IP) injection (the MetS + OVX + EGCG) group, and (e) MetS induced with the HFHS dieting combined with EGCG (10 umol/kg/day) IP injection (the MetS + EGCG) group. The major ingredients of HFHS diet (DyEts Inc, USA; DyEt, # 102829) included casein (200 gm/kg; 716 kcal/kg), soybean oil (25 gm/kg; 225 kcal/kg), and fructose (575 gm/kg; 2300 kcal/kg). The fat content of the HFHS diet was predominantly saturated.

Both ovaries were excised through bilateral abdominal incisions under halothane anesthesia (3% induction followed by 1% maintenance) to induce menopause, and every effort was made to minimize suffering and the number of animals used throughout the experiment. After OVX, the rats were allowed to recovery for two weeks, and then received HFHS diet feeding combined with/without EGCG IP injection during the following 6-month period^5^. EGCG (CAS registry number: 989–51–5) was purchased from Sigma Chemical Co. (St. Louis, MO).

### Evaluation of biochemical and estrogen hormonal parameters

Blood samples were collected from the tail artery for estrogen hormonal analysis. Estrogen hormonal analysis was assessed as described previously^6^ with minor modifications. Blood was separated by centrifugation at 4 °C. The microtiter wells of the 17-β estradiol ELISA kit (Cayman Chemical Co., Ann Arbor, MI, USA) were coated with an antibody directed towards a unique antigenic site on the estradiol molecule. After addition of the substrate solution, the intensity of color developed was inversely proportional to the concentration of estradiol in the rat sample as measured by ELISA (Bio-Tek ELX 800, BioTek, Bad Friedrichshall, Germany)^5^. The mean absorbance values for each set of standards and serum samples of experimental rats were calculated.

Blood was harvested from heart at the termination of the experiment for biochemical data. Serum activity of glutamate oxaloacetate transaminase (GOT) and glutamate pyruvate transaminase (GPT) and the concentrations of triglycerides, cholesterol, low-density lipoprotein (LDL), high-density lipoprotein (HDL), glucose, and insulin were determined by using an automated analyzer (Selectra Junior Spinlab 100, Vital Scientific, Dieren, Netherlands; Spinreact, Girona, Spain) according to the manufacturers’ instructions^20^. Total protein in urine was measured by colorimetric assay, using Pyrogallol red as dye-binding (Wako Diagnostics and Chemicals USA Inc.).

### Physical indicator, metabolic cage study for micturition pattern and cystometrogram studies

During the experimental periods, physical indicator, including body weight, waist circumference, systolic pressure, diastolic pressure and mean arterial pressure (MAP, calculated as 1/3 systolic pressure + 2/3 diastolic pressure) were measured monthly. Metabolic cage and cystometrogram studies were assessed as described previously^6,21^ with minor modifications. After 6 months of treatment, rats were placed in individual metabolic cages (R-2100; Lab Products, Rockville, Maryland). The 24-hour micturition frequency and voided volume were determined using a cup especially fitted to a transducer (MLT 0380, ADI Instruments, Colorado Springs, CO, USA). In the same time, the volume of water intake and urine output were measured. The cystometrograms (CMGs) were performed as previously described^22^. Each experimental rat was anesthetized with Zoletil50 (1 mg/kg IP). The bladder catheter (PE50 tube) was connected to both a syringe pump (KD Scientific 100, KD Scientific, Holliston, MA, USA) and a pressure transducer (MLT 0380, ADI Instruments, Colorado Springs, CO, USA).

Before the beginning of each CMG, the bladder was emptied and saline was infused at a steady rate (0.08 ml/min), during which pressure was measured via a small-volume pressure transducer in line with the catheter. A voiding contraction defined as an increase in bladder pressure that resulted in urine loss. The CMG was measured at least five filling/voiding cycles in each rat until the bladder pressure was stable. Pressure and force signals were amplified (ML866 PowerLab, ADI Instruments), recorded on a chart recorder and digitized for computer data collection (Labchart 7, ADI Instruments, Windows 7 operating system). CMG parameters recorded for each animal included micturition pressure, micturition interval, voiding volume, and non-voiding contractions.

### Histological study by Masson’s Trichrome Stain

After cystometric studies, experimental rats were perfused with saline solution through the left ventricle, and the bladders were removed to record weight and cut open in a sagittal direction. Histolgical study was performed as described previously^6,21^ with minor modifications. The bladder tissue samples from different groups were embedded in paraffin blocks, and serial sections of 5 μm thickness were obtained. Deparafinized sections were stained with Masson’s trichrome stain (Masson’s Trichrome Stain Kit, DAKO, Glostrup, Denmark). The standard Masson’s trichrome staining procedure was followed to stain collagen in connective tissue in blue and detrusor smooth muscle (DSM) in red^5,6^. Histological slices were blinded to group allocation. The bladder sections of each specimen were captured by digital camera in ten random, non-overlapping frames at 400X magnification and compared between experimental groups. The color setting and image-associated quantification were determined by image analysis software (Image-Pro Plus, Media Cybernetics, MD, and USA)^5^.

### Apoptotic cell staining by terminal deoxynucleotidyl transferase - mediated dUTP-biotin nick-end labeling (TUNEL) assay

Apoptotic cell staining was assessed as described previously^6,21^ with minor modifications. In order to detect cells undergoing apoptosis, tissue sections were processed by the TUNEL assay using the *In situ* cell death detection kit (Roche, Pleasanton, CA). The DNA strand breaks could be identified by labeling free 3′-OH with fluorescence in dUTP plus terminal transferase. The number of TUNEL-positive cells in 10 randomly selected non-overlapping fields of 400 magnifications in the bladder was calculated and compared between experimental groups. In each experiment, negative controls with label solution (without terminal transferase) instead of TUNEL reaction mixture were used to elucidate nonspecific immunostaining.

### Protein isolation and western blot analysis

Protein analysis was performed as described previously^6,21^ with minor modifications. According to the previously described method^6^, the frozen tissues of bladders were homogenized on ice in the buffer (50 mM Tris, pH 7.5, 5% Triton-X100) containing the halt protease inhibitor cocktail (Pierce, Rockford, IL, USA) at 100 mg/ml. 50 µg of protein from the bladders was loaded on sodium dodecyl sulfate (SDS) polyacrylamide electrophoresis gels and transferred to polyvinylidene fluoride membranes (Immobilon-P, Millipore, MA). Immobilon-P membranes were incubated with primary antibodies to the following markers, receptors, proteins and enzyme complexes.

Primary antibodies included CHRM2 (M2; Epitomics, Burlingame, CA, mouse monoclonal IgG1, 1:1000; MW ~52 kDa) (Clone MIgG51–4) (Catalog no.: 3021–1), CHRM3 (M3; Alomone Labs, rabbit polyclonal IgG, 1:500; MW ~66 kDa) (Catalog no.: AMR-006), P2X3 (Novus, rabbit polyclonal IgG, 1:1000; MW ~61 kDa) (Catalog no.: NB100–1658), TGF-β1 (R & D, Minneapolis, MN, rabbit polyclonal IgG1, 1:1000; MW ~15 kDa) (Catalog no.: MAB240), Fibronectin (Millipore, mouse monoclonal IgG, 1:1000; MW ~15 kDa) (Clone DH1) (Catalog no.: MAB1940), type 1 collagen (Abcam, Cambridge, MA, rabbit polyclonal IgG, 1:1000; MW ~15 kDa) (Catalog no.: ab292), Nitrotyrosine (Enzo, Farmingdale, NY, mouse monoclonal IgG1, 1:1000; MW ~95 kDa) (Clone NOY-7A5) (Catalog no.: ALX-804–208), 2,4-dinitrophenol (DNP; Bethyl, goat polyclonal IgG, 1:1000; MW ~95 kDa) (Catalog no.: A150–117A), GRP78 (Proteintech, Chicago, IL, rabbit polyclonal IgG, 1:1000; MW ~78 kDa) (Catalog no.: 11587–1-AP), CHOP (Abcam, Cambridge, MA, mouse monoclonal IgG2b, 1:1000; MW ~30 kDa) (Clone 9C8) (Catalog no.: ab11419), Caspase-12 (Abcam, Cambridge, MA, rabbit polyclonal IgG, 1:1000; MW ~40 kDa) (Catalog no.: ab18766), Bax (Proteintech, Chicago, IL, rabbit monoclonal IgG, 1:1000; MW ~24 kDa) (Catalog no.: 50599–2-Ig), Bcl-2 (Cell Signaling, Danvers, MA, rabbit monoclonal IgG, 1:1000; MW ~26 kDa) (Clone 50E3) (Catalog no.: 2870), Cytochrome c (Abcam, Cambridge, MA, mouse monoclonal IgG, 1:1000; MW ~15 kDa) (Clone 7H8.2C12) (Catalog no.: ab13575), Caspase-3 (Abcam, Cambridge, MA, rabbit polyclonal IgG, 1:500; MW ~32 kDa) (Catalog no.: ab44976), Caspase-9 (Millipore, Billerica, MA, rabbit monoclonal IgG, 1:2000; MW ~46 kDa) (Catalog no.: 04–443), NDUFS3 (Abcam, Cambridge, MA, mouse monoclonal IgG1, 1:1000; MW ~30 kDa) (Clone 3F9DD2) (Catalog no.: ab110246), SDHA (Abcam, Cambridge, MA, mouse monoclonal IgG1, 1:1000; MW ~70 kDa) (Clone 2E3GC12FB2AE2) (Catalog no.: ab14715), UQCRC2 (Abcam, Cambridge, MA, mouse monoclonal IgG1, 1:1000; MW ~48 kDa) (Clone 13G12AF12BB11) (Catalog no.: ab14745), COX-2 (Cayman, Ann Arbor, MI, mouse monoclonal IgG1, 1:1000; MW ~72 kDa) (Clone CX 229) (Catalog no.: 160112), ATPB (Abcam, Cambridge, MA, mouse monoclonal IgG1, 1:1000; MW ~52 kDa) (Clone 3D5) (Catalog no.: ab14730), and β-actin (Millipore, Billerica, MA, mouse monoclonal IgG2b, 1:1000; MW~43 kDa) (Clone C4) (Catalog no.: MAB1501).

Muscarinic (M2 and M3) and purinergic (P2X3) receptors, inflammatory fibrosis markers (TGF-β, Fibronectin, and Collagen I), oxidative stress markers (2,4 dinitrophenol (DNP) and nitrotyrosine), ER markers (GRP 78, CHOP and Caspase-12), anti-apoptotic (Bcl-2), pro-apoptotic proteins (Bax, cytochrome c, caspase-3, and Caspase-9), and the expressions of mitochondrial respiratory enzyme complexes (NDUFS3, SDHA, UQCRC1, COX-2, and ATPB) were normalized with β-actin. In each experiment, negative controls were performed without the primary antibody. For each group, there were 10 samples used and each sample was run in triplicate number of experiments.

### Immunofluorescence studies of M2 and Cox-2 expression

The bladder sections were then double stained with the primary antibodies to muscarinic acetylcholine receptor M2 (1:100, rabbit monoclonal IgG; Epitomics) and Neurofilament (1:200, mouse monoclonal IgG2b; Novus) at 4 °C overnight, then incubated with secondary antibody (1:800; Invitrogen) conjugated to fluorescein isothiocyanate (FITC) for M2, conjugated to rhodamine for Neurofilament. The nuclei of the cells were counterstained with DAPI.

### Statistical analysis

Analysis of variance, followed by the post hoc Bonferroni test and two-way analysis of variance for individual comparison, was conducted for the above experiments. The mean, standard deviation (SD), and p values were calculated on triplicate independently experiments. Student’s t-test was used to calculate p-values for comparison. The significant level was set at a p-value < 0.05.

The goals in our statistical analysis were briefly described as the following, (1) To investigate the cellular mechanism of bladder overactivity after MetS, we compared the OVX treatment with the control group, and the bladder repair after EGCG pretreatment with the control group. Statistics showed as means ± SD; the annotations of *P< 0.05 and **P < 0.01 indicated the significance of each group versus the control group. (2) To investigate potential mechanisms underlying the oxidative stress and apoptosis mediated by EGCG, we compared the EGCG group with the MetS group. The annotations of ^†^P < 0.05 and ^††^P < 0.01 indicated the significance of each group versus the MetS group. (3) To evaluate the effect of EGCG on bladder repair after OVX treatment in the MetS condition, we compared the MetS + OVX group with the MetS + OVX + EGCG group. The annotations of ^#^P < 0.05 and ^##^P < 0.01 indicated the significance of the MetS + OVX group versus the MetS + OVX + EGCG group. All statistical tests that have been performed were reported, including those that did not yield statistical significance.

## Results

### Serum estradiol concentration reduced after ovariectomy (OVX)

OVX was applied to mimic the physiological conditions of the ovarian hormone deficiency to induce bladder overactivity in rat model. Table 1 showed that the serum estradiol concentration was significantly decreased two weeks after OVX treatment: 17.9 ± 4.4 pg/ml for the MetS + OVX group and 20.9 ± 3.6 pg/ml for the MetS + OVX + EGCG group, in comparison with 42.3 ± 5.5 pg/ml, 37.8 ± 5.2 pg/ml and 42.8 ± 6.6 pg/ml for the control, the MetS and the MetS + EGCG groups, respectively.

**Table 1:** General physical indicators, urine serum biochemistry and urodynamic parameters for the different experimental groups.

	Control	MetS	MetS + OVX	MetS + OVX + EGCG	MetS + EGCG
**No**. **rats**	12	10	10	10	10
Serum estradiol conc. (pg/ml) before treatment	40.9 ± 4.0	36.8 ± 5.0	37.7 ± 5.1	37.6 ± 6.0	38.7 ± 5.6
Serum estradiol conc. (pg/ml) after treatment	42.3 ± 5.5	37.8 ± 5.2	17.9 ± 4.4**	20.9 ± 3.6**	42.8 ± 6.6
**Physical indicators**					
Body weight (gm)	380.2 ± 41.2	419.8 ± 38.8*	550.0 ± 46.4** ^††^	506.2 ± 39.4** ^††^	390.5 ± 41.1^†^
Waist circumference (cm)	18.6 ± 3.3	20.6 ± 2.8*	27.7 ± 3.4** ^†^	25.3 ± 3.1** ^†^	20.7 ± 2.7
Systolic pressure (mmHg)	116.6 ± 6.1	130.8 ± 4.6**	140.3 ± 8.0**	130.4 ± 6.4**	121.2 ± 17.8^†^
MAP (mmHg)	96.3 ± 3.0	105.0 ± 4.5*	107.9 ± 5.4*	104.4 ± 7.2*	98.6 ± 6.1^†^
**Urine parameters**					
Water intake (ml/24hrs)	30.6 ± 4.6	30.5 ± 4.1	34.4 ± 4.7	32.6 ± 5.7	29.2 ± 4.3
Urine output (ml/24hrs)	16.8 ± 3.6	18.7 ± 3.8	17.4 ± 4.2	15.8 ± 2.8	14.9 ± 3.0
Bladder weight (mg)	145.8 ± 14.4	168.8 ± 25.2*	229.6 ± 33.7** ^††^	192.8 ± 43.4** ^† #^	153.7 ± 22.6* ^†^
The ratio of bladder weight (mg) / body weight (g)	0.383 ± 0.035	0.402 ± 0.065	0.419 ± 0.072*	0.381 ± 0.044	0.393 ± 0.055
Glucose (mg/dl)	0	18.2 ± 4.3**	37.4 ± 6.3** ^†^	21.8 ± 2.7** ^† ##^	2.4 ± 0.39* ^††^
Urine protein (mg/dl)	0	17.8 ± 2.7**	53.9 ± 13.1** ^††^	20.8 ± 2.8** ^† ##^	6.6 ± 2.3** ^†^
Urine protein /Creatinine	0	53.3 ± 7.3**	302.5 ± 41.5** ^††^	73.7 ± 10.7** ^† ##^	16.8 ± 2.9** ^††^
**Serum parameters**					
GOT (U/dl)	44.6 ± 5.2	137.6 ± 28.7**	237.7 ± 49.1** ^††^	149. 5 ± 30.6** ^† ##^	51.7 ± 9.3* ^†^
GPT (U/dl)	25.5 ± 4.5	51.6 ± 10.3**	80.9 ± 24.4** ^††^	65.1 ± 9.2** ^† #^	32.3 ± 7.3* ^†^
Triglycerides (mg/dl)	78.6 ± 13.4	149.7 ± 20.2**	235.7 ± 36.3** ^††^	163.5 ± 28.9** ^† ##^	87.3 ± 18.6^†^
Cholesterol (mg/dl)	66.2 ± 11.5	186.7 ± 35.9**	366.5 ± 45.5** ^††^	215.1 ± 34.8** ^† ##^	79.9 ± 15.7** ^†^
HDL (mg/dl)	31.7 ± 3.2	28.0 ± 3.9*	24.6 ± 4.2** ^†^	27.3 ± 4.2*	32.3 ± 5.4^†^
LDL (mg/dl)	10.3 ± 2.8	50.7 ± 9.8**	87.2 ± 16.8** ^††^	59.8 ± 8.5** ^† ##^	19.6 ± 3.4* ^††^
Glucose (mg/dl)	125.0 ± 9.4	156.5 ± 21.9**	175.6 ± 25.4** ^††^	158.7 ± 18.2** ^#^	132.2 ± 20.4^†^
Insulin (Bayer) (mU/L)	0.5 ± 0.02	0.5 ± 0.06	0.5 ± 0.04	0.5 ± 0.05	0.5 ± 0.06
LDH	125.6 ± 15.2	383.0 ± 67.9**	1373.0 ±± 168.5** ^††^	475.8 ± 57.0** ^† ##^	186.3 ± 37.7* ^†^
**Urodynamic parameters**					
Frequency (No. voids /1 hr)	4.2 ± 1.5	7.6 ± 2.8**	12.7 ± 3.5** ^††^	7.6 ± 2.1** ^##^	5.4 ± 1.5* ^†^
Peak micturition pressure (cmH2O)	32.3 ± 2.0	40.6 ± 4.9*	53.5 ± 5.1** ^††^	40.0 ± 4.7* ^##^	33.8 ± 3.5^††^
Voided volume (ml)	2.4 ± 0.5	1.5 ± 0.2**	0.8 ± 0.3** ^††^	1.6 ± 0.3** ^##^	1.8 ± 0.4* ^†^
No. of non-voiding contractions between micturition (No./hr)	0	0	3.0 ± 0.7** ^††^	0 ^##^	0

Footnote: OVX, surgical ovariectomy; EGCG, epigallocatechin gallate. MAP, mean arterial pressure; GOT, glutamate oxaloacetate transaminase; GPT, glutamate pyruvate transaminase; LDL, low-density lipoprotein; HDL, high-density lipoprotein; LDH, Lactic dehydrogenase; Valus are means ± SD. *P < 0.05; **P < 0.01 versus the control group. ^**†**^P < 0.05; ^**††**^P < 0.01 versus the MetS group. ^#^P < 0.05; ^##^P < 0.01, the MetS + OVX group versus the MetS + OVX + EGCG group.

### Functional analysis on physical indicators and biochemical parameters

It is well recognized that MetS is characterized by hypertension, dyslipidemia, abdominal obesity and insulin resistance. After feeding with HFHS diet for 6 months, the rats developed MetS as shown in physical indicators and biochemical parameters. General physical indicators, including body weight, waist circumference, systolic pressure, mean arterial pressure (MAP) were present in Table 1. The physical indicators of MetS developed in the MetS, the MetS + OVX and the MetS + OVX + EGCG groups revealed significant increases in body weight, waist circumference, systolic pressure and MAP, as compared to the control group.

Additionally, there was no significant difference in the amount of water intake and urine output among different groups. However, the MetS, the MetS + OVX and the MetS + OVX + EGCG groups showed significant increases in bladder weight, urine glucose, urine protein and the ratio of urine protein to creatinine, as compared to the control group. Especially, the MetS combined with OVX group resulted in significant renal function deterioration. Such alterations of physical indicators and biochemical parameters were reversed when the MetS + EGCG group was compared with the MetS group.

Similarly, the serum parameters associated with the symptoms of MetS, including GOT, GPT, triglycerides, cholesterol, LDL, glucose and LDH (except insulin level), were significantly elevated in the MetS, MetS + OVX and MetS + OVX + EGCG groups, as compared to the control. Pretreatment with EGCG reversed the levels of serum triglycerides, HDL and glucose in the MetS + EGCG group to almost the control level. However, there was a limitation on the EGCG pretreatment for MetS + OVX + EGCG group to restore to the control level. These results indicated that metabolic abnormalities in combination with ovarian hormone deficiency caused a profound negative effect on biochemical parameters, resulting in irreversible changes.

### Bladder function and voiding behavior

**Figure 1:**
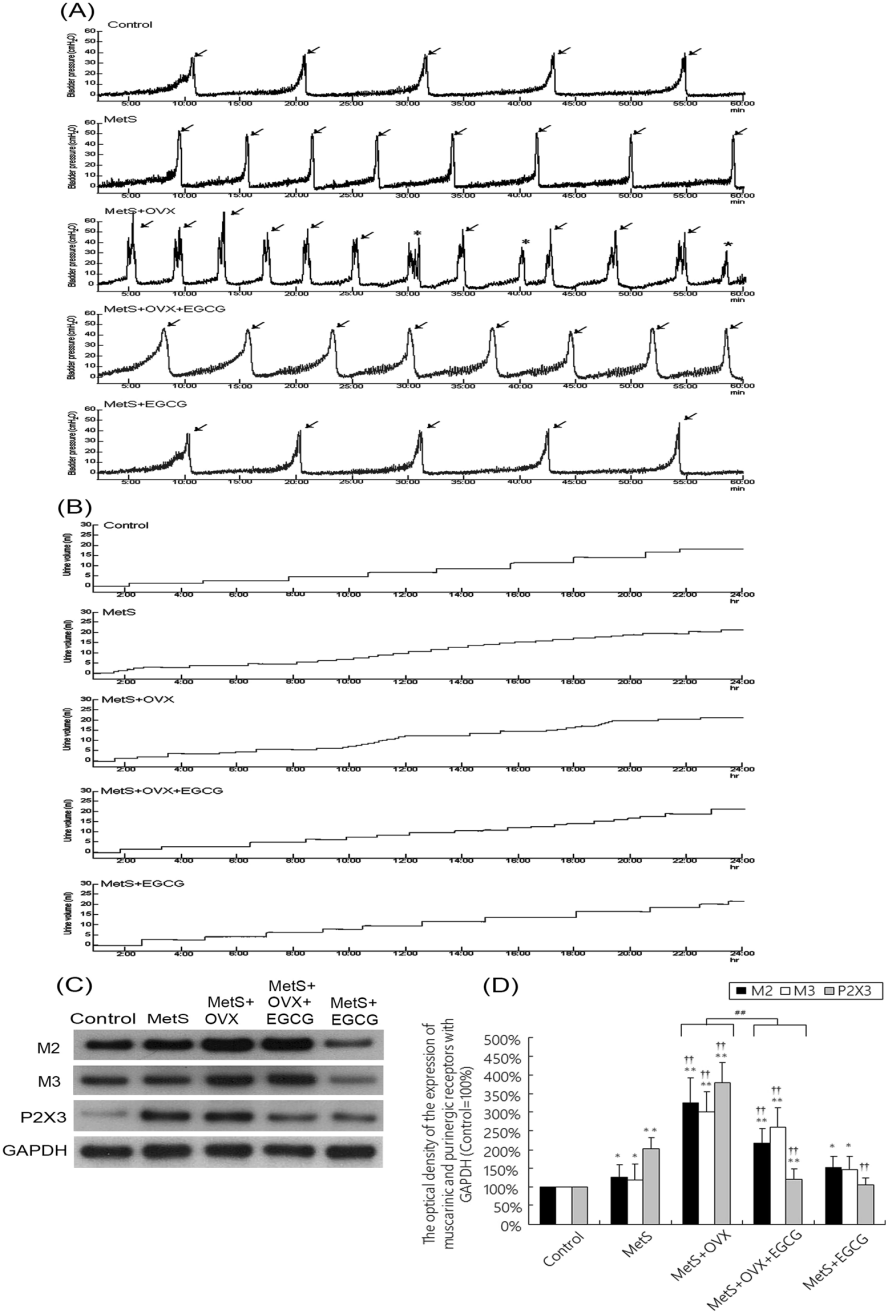
Bladder cystometric parameters and voiding behavior in a rat model with MetS and ovarian hormone deficiency. (**A**) Cystometry recordings illustrating micturition pressure, voiding frequency, voiding contractions (arrows) and non-voiding contractions (stars). (**B**) Tracing analysis of 24 hours voiding behavior by metabolic cage. The recordings showed that the MetS + OVX group significantly increased bladder maturation pressure, voiding contractions, non-voiding contractions and micturition frequency than the other groups. (**C**,**D**) Western blots of the bladder muscarinic (M2 and M3) and purinergic (P2X3) receptors. The receptor expressions were significantly increased in the MetS + OVX group. However, administration with EGCG significantly decreased the expression levels of these receptors in the MetS + OVX + EGCG group. Values were the mean ± SD for n = 8. ^*^P < 0.05; ^**^P < 0.01 versus the control group. ^**†**^P < 0.05; ^**††**^P < 0.01 versus the MetS group. ^##^P < 0.01, the MetS + OVX group versus the MetS + OVX + EGCG group.

We investigated the pathophysiological relationship between MetS and bladder overactivity. Table 1 and Fig. 1 reported the urodynamic parameters and the voiding behavior analysis by metabolic cage, including micturition frequency, micturition pressure, voided volume, and non-voided contraction between micturition. The MetS, MetS + OVX and MetS + OVX + EGCG groups increased micturition frequency and peak micturition pressure, but decreased micturition interval and bladder volume, as compared with the control group. Moreover, CMG study and voiding behavior analysis demonstrated that the MetS + OVX group enhanced micturition frequency, micturition pressure and non-voiding contractions (stars), but reduced voiding volume, as compared with the MetS group (Fig. 1A,B and Table 1).

However, EGCG pretreatment in the MetS + OVX + EGCG group significantly lessened the voiding pressure and non-voiding contractions compared to the MetS + OVX group. These results implied that ovarian hormone deficiency exacerbated MetS rats deteriorated bladder overactivity and cause abnormal detrusor activity. However, EGCG efficacy slightly improved, but not fully recovered from such deteriorations as shown in the MetS + OVX + EGCG group.

### The expressions of muscarinic and purinergic receptors

At the surface of bladder urothelium, various receptors, including muscarinic (M2 and M3) and purinergic (P2X3), increased bladder afferent nerve activity and detrusor activity. Thus, urothelial receptors likely contributed to therapeutic efficacy for lower urinary tract symptoms. Figure 1C,D presented the expressions of M2, M3 muscarinic and P2X3 purinergic receptors as shown by western blotting and their statistical comparisons. In comparison with the control, the expressions of muscarinic (M2 and M3) and purinergic (P2X3) receptors were increased in the MetS, the MetS + OVX and the MetS + OVX + EGCG groups. Especially, the expressions in the MetS + OVX group were notably enhanced compared with the MetS group and the MetS + OVX + EGCG group. In Fig. 1C,D, EGCG pretreatment showed the ability of reducing the extent of such over-expressions in the MetS + OVX + EGCG group versus the MetS + OVX group.

To further explore the bladder overactivity after MetS and OVX treatment and the bladder repair after EGCG pretreatment, the localization of M2 (red) and Neurofilament (green) double immunoreactivity was assessed for urothelial layer (UL) and suburothelial layer (SL) (Supplementary Fig. 1). In the control group, the M2 expression was slightly co-stained with neurofilament and mainly distributed in the basal layers of the urothelium (yellow arrows) (Supplementary Fig. 1A). However, in the MetS, the MetS + OVX and the MetS + OVX + EGCG groups, the distribution of the M2 staining (white arrows) was enchanced throughout the thinner and distrupted UL (white arrowheads) and the lamina propria of the SL. Moreover, the immunostaining of M2 (white arrows) was decreased in the MetS + EGCG group as compared to the MetS + OVX + EGCG group.

On the other hand, the localization of M2 (red) and neurofilament (green) double immunoreactivity (arrows) was examined the muscular layer (ML) (Supplementary Fig. 2). M2 and neurofilament proteins were strongly colabeled in the neural gangion (arrowheads) between detrusor smooth muscle (DSM) bundles in the control and the MetS groups. However, after MetS and OVX treatment, the co-labeling of M2 and neurofilament (arrows) was significantly decreased. These results revealed that MetS and OVX treatment strongly induced the receptor expression of M2 in defected urothelial lining. While, EGCG pretreatment improved the bladder repair.

### Bladder histological features in association with MetS and ovarian hormone deficiency

**Figure 2:**
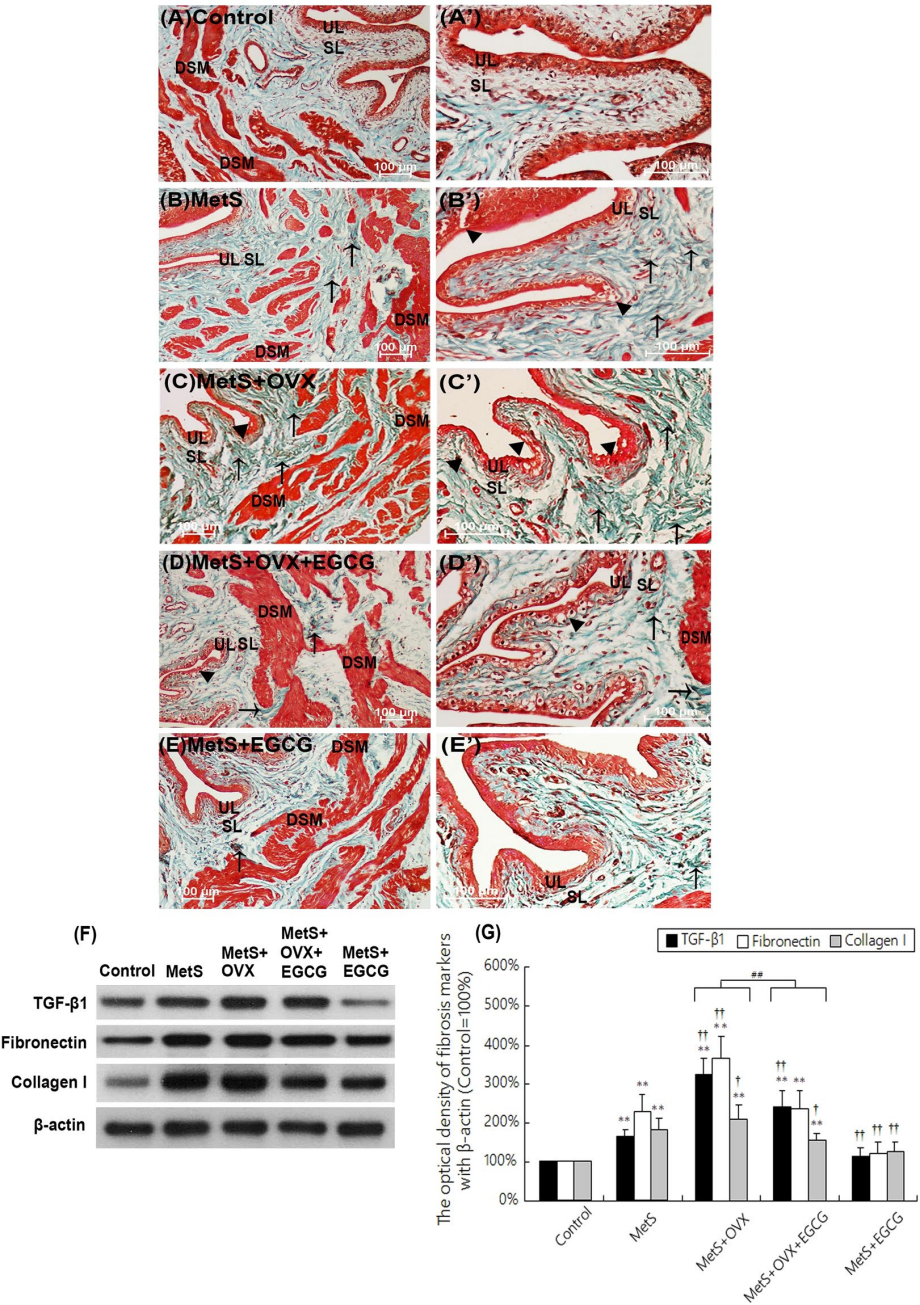
The bladder pathological features of the MetS group induced by diet and ovarian hormone deficiency incurred by ovariectomy, as shown by Masson’s trichrome staining and fibrosis marker expressions. (**A**–**E**) Masson’s trichrome stain showed the blue-stained collagen and the red-counterstained DSM highlighted for each image. In the control group (**A** and A’), there were 3–5 layers of urothelial layer (UL) and only sparse collagen distributing in suburothelial layer (SL). In contrast, there were significant interstitial fibrosis and collagen accumulation (arrows) between detruser smooth muscle (DSM) bundles in MetS group (**B** and B’), MetS + OVX group (**C** and C’), and MetS + OVX + EGCG group (**D** and D’). The MetS + OVX group revealed the increased bladder fibrosis (arrows), denuded urothelial mucosa (arrowheads), and thining UL. (**F**,**G**) Western blots for fibrosis marker expression were measured and probed with an antibody specific to TGF-β, fibronectin and type I collagen in each group. Quantifications of the percentage of TGF-β, fibronectin and type I collagen expressions to β-actin were shown. Results were normalized as the control = 100%. The expressions of TGF-β, fibronectin and types I collagen were significantly increased in the MetS, MetS + OVX, and MetS + OVX + EGCG groups. However, EGCG administration greatly decreased fibrosis marker expression. (**A**–**E**), magnification X200; A’–E’, magnification X400. Scale bar = 100 μm. Values were the mean ± SD for n = 8. ^**^P < 0.01 versus the control group. ^**†**^P < 0.05; ^**††**^P < 0.01 versus the MetS group. ^##^P < 0.01, the MetS + OVX group versus the MetS + OVX + EGCG group.

Masson’s trichrome stain was performed to investigate the pathological changes in the bladder after the treatment of HFHS dieting, bilateral ovariectomy or EGCG. Figure 2 revealed that in the control group (Fig. 2A,A’), there were 3–5 layers of urothelial layer (UL) and only sparse collagen distributing in suburothelial layer (SL). Similarly, there was only sparse collagen accumulation between detrusor smooth muscle (DSM) bundles in the muscular layer. On the contrary, there was significant interstitial fibrosis and collagen accumulation (arrows) between DSM bundles in the MetS (Fig. 2B,B’), MetS + OVX (Fig. 2C,C’) and MetS + OVX + EGCG (Fig. 2D,D’) groups. Moreover, the MetS + OVX group (Fig. 2C,C’) showed denuded urothelial mucosa as well as defective and thining urothelium (black arrowhead) and also significant interstitial fibrosis (arrows), as compared to the Met and MetS + OVX + EGCG groups (Fig. 2D,D’). However, bladder tissues in the MetS + EGCG group exhibited significant reduction in interstitial fibrosis and collagen accumulation, in comparison with the MetS group (Fig. 2E,E’).

The expressions of inflammatory and fibrosis markers (TGF-β, fibronectin and type I collagen) were examined by western blotting (Fig. 2F,G). In comparison with the control, the expression of TGF-β, fibronectin and type I collagen proteins were noticeably enhanced in either the MetS group versus the MetS + EGCG group, or the MetS + OVX group versus the MetS + OVX + EGCG group. After EGCG pretreatment, these expressions were significantly decreased as shown in the MetS + EGCG group as compared to the MetS group and the MetS + OVX + EGCG group as compared to the MetS + OVX group.

These observations revealed that the expressions of inflammatory and fibrosis markers were significantly enhanced in the MetS and MetS + OVX groups, indicating an increase in bladder interstitial fibrosis. Nevertheless, the administration of EGCG alleviated the interstitial fibrosis and collagen accumulation between DSM bundles in the bladders.

### Expressions of oxidative markers in the bladder with MetS and ovarian hormone deficiency

**Figure 3:**
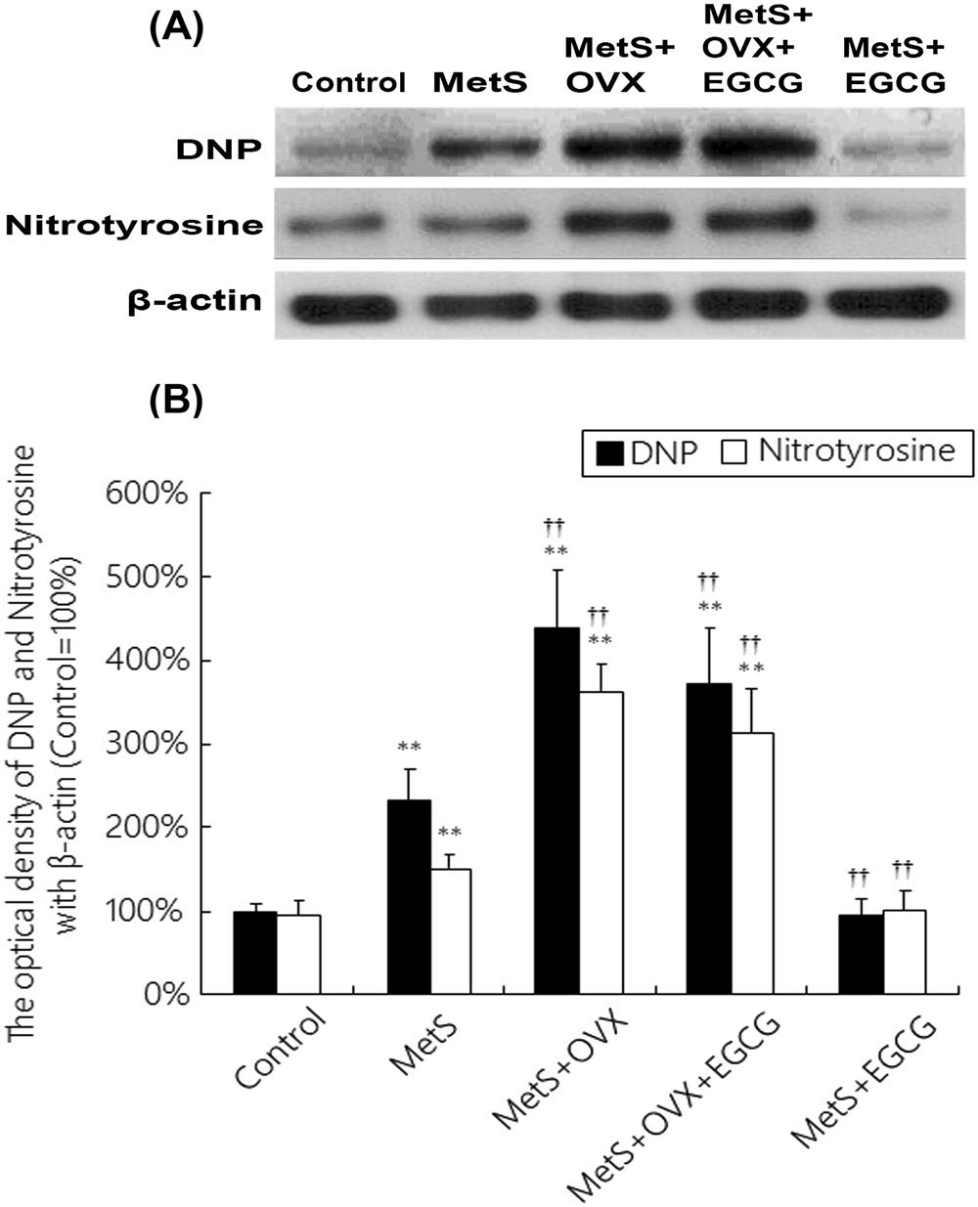
The effects of EGCG on the expressions of oxidative stress markers in the status of MetS and ovarian hormone deficiency. (**A**) The expression levels of oxidative stress markers (DNP and nitrotyrosine) by western blots. (**B**) Quantifications of the percentage of DNP and nitrotyrosine to β-actin in different experimental groups. The expression levels were slightly increased in the MetS group, and significantly enhanced in the MetS + OVX and MetS + OVX + EGCG groups. Results were normalized as the control = 100%. Values represented the mean ± SD for n = 8. ^**^P < 0.01 versus the control group. ^**††**^P < 0.01 versus the MetS group.

Figure 3 showed that the expressions of oxidative stress markers (DNP and nitrotyrosine) as shown by western blots were significant overexpressed in the MetS, MetS + OVX and MetS + OVX + EGCG groups in comparison with the control. The expression levels of DNP as well as nitrotyrosine were 2.4-fold as well as 1.6-fold of control in the MetS group, and 4.3-fold as well as 3.6-fold of control in the MetS + OVX group.

After EGCG pretreatment, the levels of DNP and nitrotyrosine were slightly reduced to 3.7-fold and 3.2-fold of control in the MetS + OVX + EGCG group, respectively. However, such levels were reduced in the MetS + EGCG group to the extent similar to the control group (Fig. 3B). Therefore, EGCG pretreatment significantly reduced the expressions of oxidative markers in the MetS group. However, there was no significantly difference between the Met + OVX group and the Met + OV + EGCG groups. The effect of MetS in combination with ovarian hormone deficiency seemed to be more complex than MetS alone, resulting in EGCG effects that were not as profound.

The above observations revealed that both MetS and ovarian hormone deficiency induced the expressions of oxidative markers in the bladder, and EGCG reduced the extent of these expressions as shown in the MetS + EGCG group. These findings demonstrated that MetS and ovarian hormone deficiency status exacerbated oxidative damage of the bladder, whereas antioxidant EGCG offered a beneficial effect on lessening MetS and ovarian hormone deficiency- related oxidative damages.

### Mitochondria and ER - elicited bladder apoptosis with MetS and ovarian hormone deficiency

**Figure 4:**
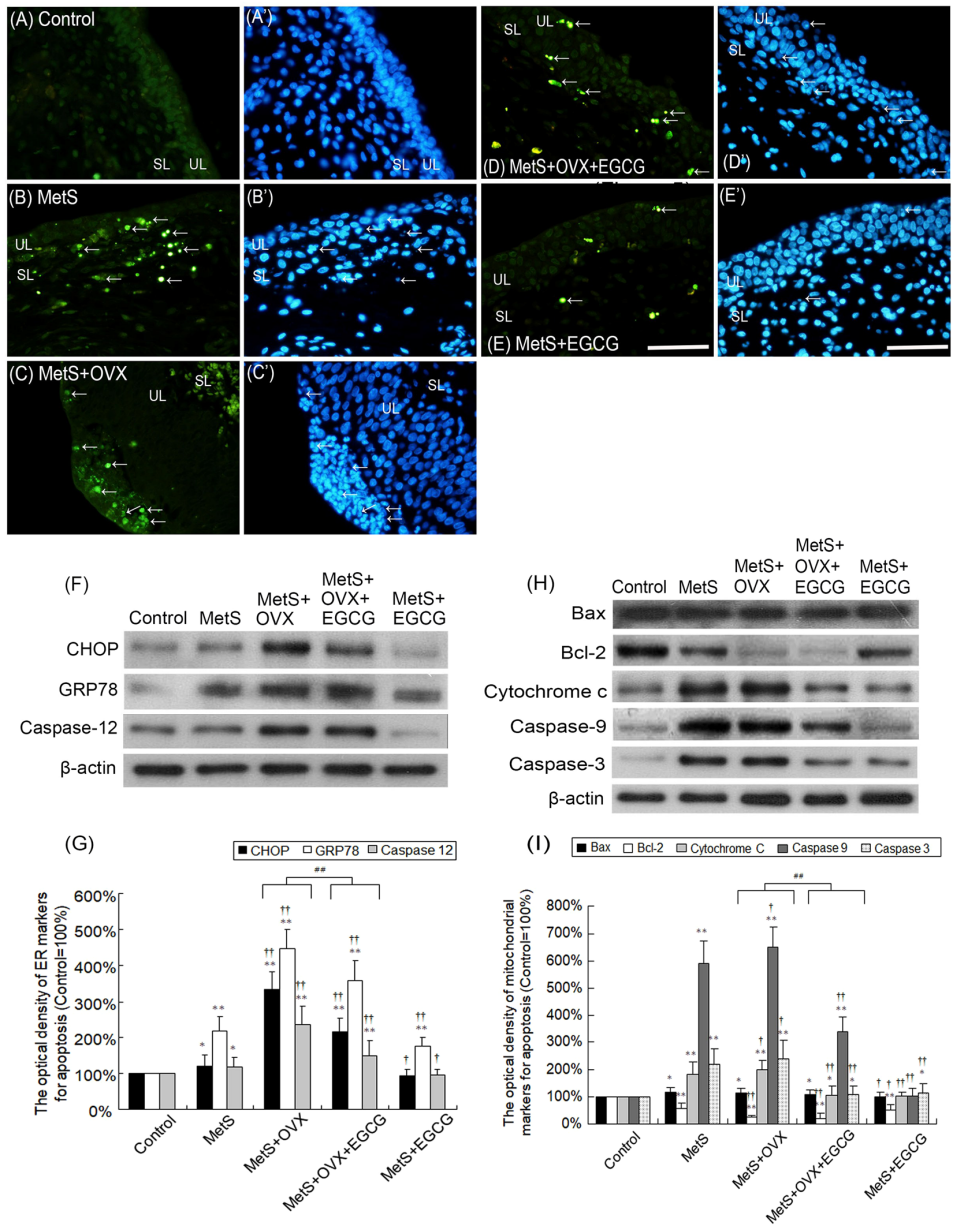
Alteration of the expressions of apoptosis-associated mitochondrial and ER stress proteins in the bladder among different experimental groups. (**A**–**E**) The apoptosis of bladder cells as detected by TUNEL, (FITC, green) and DAPI staining (blue) for UL and SL. There were increases in TUNEL - positive nuclei (white arrows) in the MetS, MetS + OVX, and MetS + OVX + EGCG groups. Scale bar = 100 μm. (**F**–**I**) The expression levels of pro-apoptotic and anti-apoptotic proteins in the bladder tissue by western blots. (**F**,**G**) Quantifications of the ER stress protein expressions to β-actin. The expression levels of GRP78, CHOP and caspase-12 slightly were increased in the MetS group, and significantly promoted in the MetS + OVX and MetS + OVX + EGCG groups. (**H**,**I**) Quantifications of mitochondrial protein expressions to β-actin. The expression of Bcl-2 was decreased in the MetS, MetS + OVX, and MetS + OVX + EGCG groups. In contrast, the pro-apoptosis expressions were meaningfully increased in those groups. Results were normalized as the control = 100%. Values represented the mean ± SD for n = 8. ^*^P < 0.05; ^**^P < 0.01 versus the control group. ^**†**^P < 0.05; ^**††**^P < 0.01 versus the MetS group. ^##^P < 0.01, the MetS + OVX group versus the MetS + OVX + EGCG group.

Figure 4 presented the results of TUNEL staining for detecting the degenerated apoptotic cells in the bladder after HFHS dieting and bilateral OVX. The apoptotic cells in the UL and SL were significantly increased in the MetS, MetS + OVX, and MetS + OVX + EGCG groups, as compared to the control group. In contrast, the apoptotic cells were noticeably decreased in the MetS + EGCG group (Fig. 4A–E). These observations revealed that ovarian hormone deficiency exacerbated MetS - related apoptosis in the bladders of rats as shown in the MetS + OVX group.

Furthermore, the involvement of ER stress signaling was evaluated by ER chaperone GRP78, ER-associated apoptosis proteins (CHOP and Caspase-12). Western blot results showed that the expression levels of CHOP, GRP78 and Caspase-12 were significantly increased by 1.3-fold, 2.2-fold and 1.3-fold of control, respectively, when the MetS group was compared with the control group. On the other hand, the MetS + OVX group enhanced the expressions of CHOP (3.4-fold of control), GRP78 (4.5-fold of control) and Caspase-12 (2.3-fold of control) in comparison with the control group. While, EGCG pretreatment lessened the expression levels of ER stress - associated proteins as shown in the MetS + OVX + EGCG versus the MetS + OVX group and the MetS + EGCG group versus the MetS group (Fig. 4F,G). These findings implied that the expression levels of ER stress signaling (CHOP, GRP78 and Caspase-12) were increased due to MetS and ovarian hormone deficiency.

Moreover, mitochondrial proteins associated with apoptosis were evaluated and the antioxidant effect of EGCG pretreatment on bladder apoptosis was investigated. Figure 4H,I indicated that the expression of anti-apoptotic protein Bcl-2 in the bladder tissues was significantly decreased in the MetS, MetS + OVX, MetS + OVX + EGCG, and MetS + EGCG groups as compared to the control group. On the contrary, the expressions of pro-apoptotic proteins (Bax, cytochrome c, caspase-9 and -3) were noticeably enhanced in the MetS, MetS + OVX, and MetS + OVX + EGCG groups. Furthermore, the MetS group enhanced the expressions of Bax (1.2-fold of control), cytochrome c (1.8-fold of control), caspase-9 (6.0-fold of control) and caspase-3 (2.1-fold of control) compared with the control group. Additionally, there was an increase in the expressions of Bax (1.1-fold of control), cytochrome c (2.0-fold of control), caspase-9 (6.4-fold of control) and caspase-3 (2.2-fold of control) in the MetS + OVX group in comparison with the control group. However, EGCG pretreatment reduced these expressions when the MetS + OVX + EGCG group was compared with the MetS + OVX group.

The above findings demonstrated that MetS and OVX induced bladder apoptosis through mitochondrial and ER-dependent apoptosis pathways. In contrast, the effect of EGCG relieved the bladder apoptosis in the status of MetS and ovarian hormone deficiency (Fig. 4H,I).

### Mitochondria respiratory enzyme complexes involving reactive oxygen species (ROS) generation

**Figure 5:**
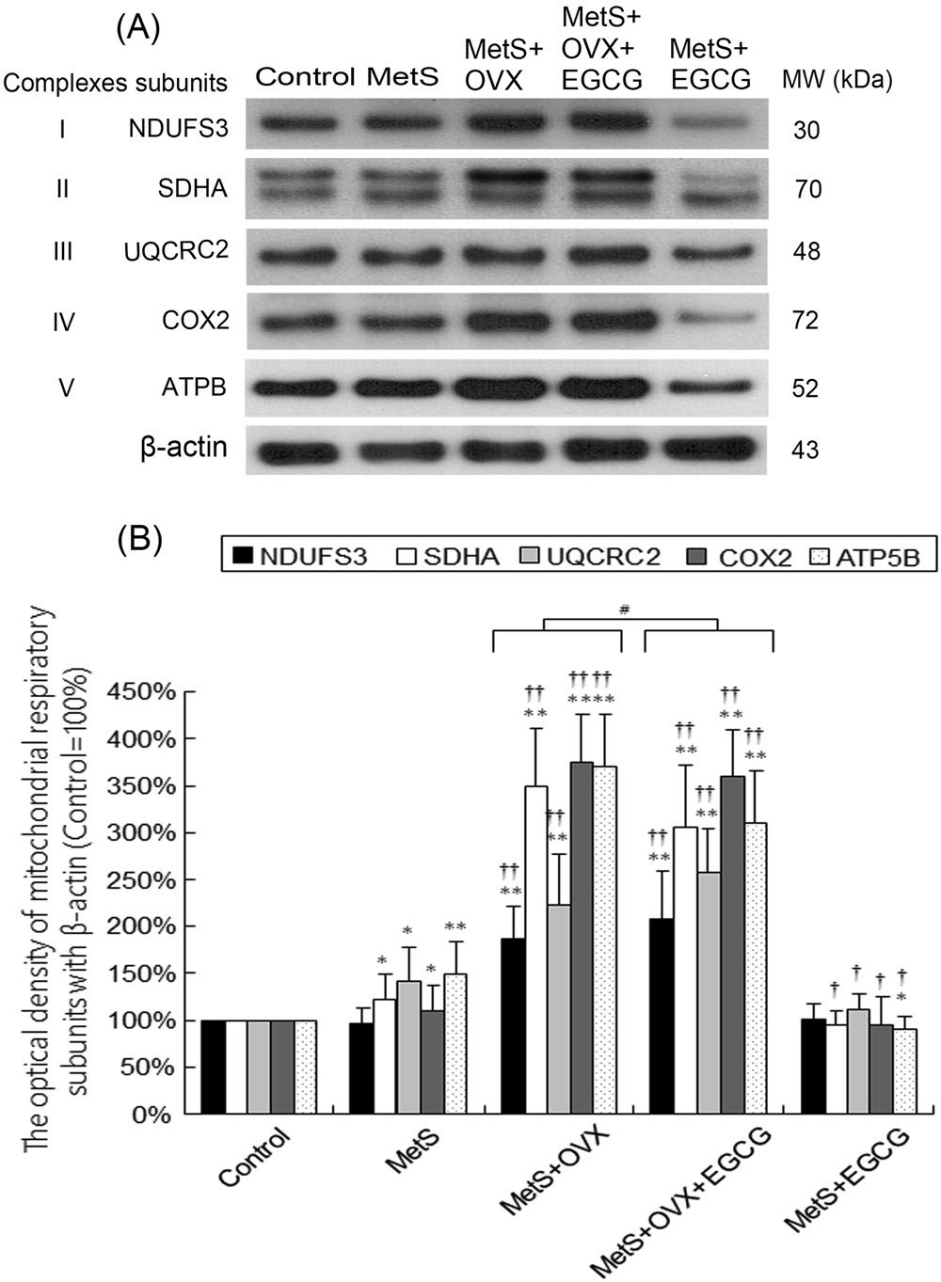
Up-regulation of the subunits of mitochondrial respiratory enzymes with MetS and ovarian hormone deficiency. (**A**) The expression levels of the subunits of mitochondrial respiratory enzymes (NDUFS3, SDHA, UQCRC1, COX-2, and ATPB) as shown by western blots. (**B**) Quantifications of the percentage of these mitochondrial respiratory enzymes to β-actin. Results were normalized as the control = 100%. The expressions of these subunits were increased in the MetS group, and noticeably enhanced in the MetS + OVX and MetS + OVX + EGCG groups. Values represented the mean ± SD for n = 8. ^*^P < 0.05; ^**^P < 0.01 versus the control group.; ^**††**^P < 0.01 versus the MetS group. ^#^P < 0.05, the MetS + OVX group versus the MetS + OVX + EGCG group.

The question whether ROS production via the mitochondrial respiratory chain was a causal link between high fat and high sugar or the main pathways responsible for bladder damage mediated by MetS and OVX ovarian hormone deficiency was studied. The assessment of mitochondrial activity was examined by the analysis of the expressions of the subunits of mitochondrial respiratory enzyme complexes involving in the generation of ROS. Western blots demonstrated that the expression levels of these enzyme subunits (NDUFS3 of complex I, SDHA of complex II, UQCRC2 of complex III, COX-2 of complex IV, and ATPB of complex V) were slightly increased in the MetS group, and were more enhanced in the MetS + OVX and MetS + OVX + EGCG groups, as compared to the control group (Fig. 5). The expressions of SDHA (1.2-fold of control), UQCRC2 (1.4-fold of control), COX-2 (1.1-fold of control) and ATPB (1.5-fold of control) were slightly promoted in comparing the MetS group with the control group. Additionally, there was an increase in the expressions of NDUFS3 (1.8-fold of control), SDHA (3.5-fold of control), UQCRC2 (2.2-fold of control), COX-2 in (3.7-fold of control) and ATPB (3.6-fold of control) in the MetS + OVX group. However, there was a decrease in the expressions in the MetS + OVX group compared with the MetS + OVX + EGCG group.

The enhancement in the expressions of mitochondrial respiratory enzyme complexes implied that MetS and ovarian hormone deficiency status could lead to generating ROS, while the extent of such increase was lessened by the pretreatment of EGCG. Comparing with the MetS group (Fig. 5B), the levels of SDHA, UQCRC1, COX-2, and ATPB were decreased in the MetS + EGCG group. Similarly, the levels of SDHA and ATPB were partially decreased after EGCG pretreatment in the MetS + OVX + EGCG group compared with the MetS + OVX group (Fig. 5B).

**Figure 6:**
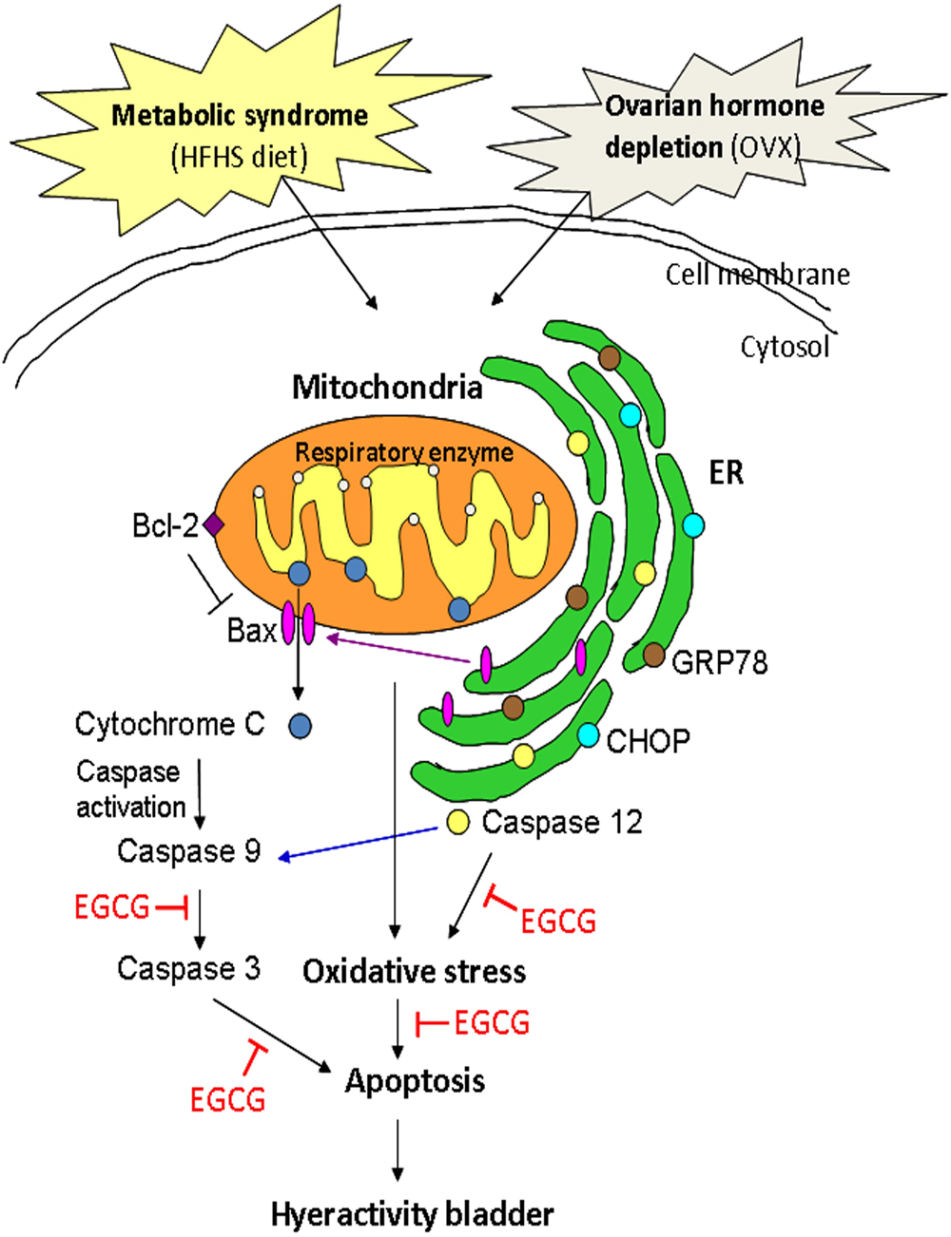
Proposed mechanistic model for MetS and ovarian hormone deficiency - induced oxidative stress through mitochondria and ER - mediated apoptosis pathways and the potential effect of EGCG on bladder overactivity. MetS and ovarian hormone deficiency induced mitochondria to release cytochrome C and caspase activation (caspase 9 and 3), and promoted ER to release GRP78, CHOP and caspase 12 to induce the generation of oxidative stress and apoptosis However, pretreatment attenuated oxidative stress induced by MetS or/and ovarian hormone deficiency, and lessened the expression of mitochondrial and ER apoptotic signals. (HFHS, high fat high sugar dieting; OVX, Ovariectomy; ER, Endoplasmic reticulum).

The above findings led to suggest a potential rat model for EGCG effect on oxidative stress and apoptosis pathway mediated through mitochondria and ER as shown in Fig. 6. The figure revealed that MetS or/and ovarian hormone deficiency induced the mitochondria to release cytochrome C and activate caspase (caspase 9 and 3), and to promote ER to release GRP78, CHOP and caspase 12. These results implied that MetS and ovarian hormone deficiency enhanced the generation of oxidative stress and apoptosis through mitochondria and ER pathways. In contrast, EGCG attenuated oxidative stress and decreased the mitochondrial- and ER-apoptotic signals, implying that EGCG alleviated bladder overactivity through mitochondria and endoplasmic reticulum-mediated apoptosis pathways in rats with MetS and ovarian hormone deficiency.

## Discussion

The present study showed that both MetS and ovarian hormone deficiency affected bladder voiding dysfunction, including increasing micturition frequency and peak micturition pressure, diminishing bladder voided volume, and raising oxidative stress, which resulted in bladder damage and interstitial fibrosis. Moreover, bladder tissue damages were accompanied by enhancement in the expressions of apoptosis-associated proteins and ER stress markers, which displayed the features of mitochondria- and ER-mediated apoptotic signals. Meanwhile, the expression levels of the subunits of mitochondria respiratory enzymes and oxidative stress markers were significantly increased in bladder tissues. The results suggested that MetS combined with ovarian hormone deficiency exhibited profound oxidative damage through mitochondria- and ER-dependent pathways, which resulted in bladder apoptosis. Through mitochondria and endoplasmic reticulum - mediated pathways, EGCG lessened oxidative stress and bladder overactivity in rats with MetS and ovarian hormone deficiency.

In previous reports, MetS was prevalent in postmenopausal women^1,2^. As a result of ovarian hormone deficiency and age-related changes, postmenopausal women are susceptible to many urological dysfunctions, including OAB symptoms, stress incontinence and recurrent urinary tract infection^1,23^. When metabolic abnormalities combined with ovarian hormone deficiency, the present study found that there were significantly bladder overactivity and interstitial fibrosis, which was compatible with clinical observations. MetS characterized with hyperlipidemia and hyperglycemia, and insulin resistance in females was related to the OAB symptoms^24–26^.

Besides, adult rats fed with high fat diet for seven weeks exhibited not only obesity, insulin resistance and hepatic steatosis, but also increased oxidative stress in liver mitochondria, suggesting that alterations in the mitochondrial compartment induced by a high fat diet were associated with the development of ectopic fat storage in the liver^27^. Our results were compatible with these findings that oxidative stress and mitochondrial dysfunction induced by metabolic perturbations would be the underlying mechanisms of bladder dysfunction. In our previous study, ovary hormone deficiency induced overactivity bladder dysfunction via intramural nerve damage and muscarinic (M2 and M3) receptor overexpression^5^. The present study found that the status of MetS and ovarian hormone induced bladder hyperactivities via up-regulations of muscarinic (M2 and M3) and purinergic (P2X3) receptors, and EGCG provided a beneficial effect by suppressing such induction.

Previous investigations suggested that EGCG was an extracted purified compound and was injected intraperitoneally^5,6^. EGCG affected mitochondrial signal transduction in a concentration-dependent manner^28^. In a lower concentration range (1–10 umoles), EGCG could inhibit the pro-apoptotic caspase, and increase the Bax gene degradation via the proteasome and protein kinase C pathways^29^. Additionally, at higher concentrations (10–50 umoles), EGCG would induce caspase-dependent apoptosis and depolarization of mitochondrial membrane to exhibit pro-oxidant and pro-apoptotic activity^30^. In our previous study, the concentration of 10 umoles EGCG was shown to exhibit strong neuroprotective, antioxidant, anti-apoptotic and anti-fibrotic effects in surgical menopause-induced overactive bladder in a rat model^5^^,^^6^. Accordingly, the concentration of 10 umoles EGCG was chosen in the present study. Proteins that directly bind with EGCG, including fibronectin and histindine-rich glycoprotein, might act as carrier proteins for EGCG^31^. However, EGCG is not only a powerful antioxidant but also a pan-assay interfering substance (PAINS); therefore, the results obtained from PAINS would be interpreted carefully^32^.

Induction of CHOP and caspase-12 involved in the ER-specific apoptosis pathways. CHOP works as a transcriptional factor that regulates genes involved in cell death. Over-expression of CHOP leads to decreasing the expression of Bcl-2 protein and inducing the translocation of Bax protein to mitochondria^33^. Moreover, caspase-12 located at ER can downstream caspases family in the cytosol in cisplatin-induced apoptosis in renal tubular epithelial cells^34^. Therefore, caspase-12 mediated the apoptosis pathway of ER, occurred as mitochondrial targeted signals^14,35^. In the present study, the increased induction of GRP78, CHOP, and caspase-12 in surgical ovarian hormone deficiency and HFHS diet - induced MetS led to apoptosis in bladders. Meanwhile, bladder tissues were accompanied by an enhancement in the expressions of apoptosis-associated proteins (Bax, cytochrome c, caspase-3, and -9), which displayed the features of mitochondria-dependent apoptotic signals. Thus, mitochondria remained an important strategy for prevention and therapy of MetS and ovarian hormone deficiency.

Increasing ROS production in the mitochondria and depleting antioxidant defenses were reported to result from hyperglycemia-induced increase in the proton gradient across the inner mitochondrial membrane. When the gradient exceeds a threshold, complex III electron transfer is blocked, leading to leakage of electrons from ubiquinone, with formation of superoxide^36^. Moreover, mitochondrial complex II dysfunction in HFHS-fed mice is associated with oxidative post-translational modifications and mitochondrial ROS could contribute to myocardial dysfunction in metabolic heart disease^37^. Other reports also indicated that the fission-mediated fragmentation of mitochondria is associated with enhanced production of mitochondrial ROS and cardiovascular cell injury in hyperglycemic conditions^38–40^. These findings suggested that mitochondrial fission is an early component that regulates mitochondrial ROS production during hyperglycemia-induced cell death.

In current clinical treatment for OAB, anti-muscarinic agents, β3-adrenoceptor agonists, and botulinum toxins intravesical injection have been reported to be helpful^41^. But these agents suppress the OAB symptoms without alleviating the underlying etiology; therefore, additional treatment modalities are needed. Pelvic ischemia in the elderly now is a new concept for voiding dysfunction by inducing oxidative stress, free radical injury to smooth muscle cells, mitochondria and ER and bladder fibrosis^42,43^. Thus, antioxidants or free radial scavengers to protect against oxidative damage could be a potential treatment of LUTS in pelvic ischemia^43^. Arthrosclerosis was associated with bladder dysfunction in rat experiment^44^. MetS is considered as an important risk factor of atherosclerosis. Additionally, ischemia injury to the bladder could occur in OVX rats^45^.

Our results revealed that MetS characterized with hyperglycemia, hyperlipidemia and hypertension could increase oxidative stress, resulting from an enhancement in the expressions of mitochondria respiratory enzyme complexes. However, ovarian hormone deficiency appeared to be a synergic effect with metabolic dysfunction, enhanced the expressions of mitochondria respiratory enzyme complexes (I to V) to spur on extensive oxidative injury in the rat bladders. These findings suggested the regulation of mitochondrial function at a transcriptional level in response to MetS and ovarian hormone deficiency.

Coyle *et al*. demonstrated that urothelial cell death via H_2_O_2_ - induced oxidative stress was mediated through superoxide, suggesting that green tea polyphenols can protect against oxidative stress/damage and bladder cell death^46^. The present results were compatible with our previous reports that EGCG pretreatment significantly increased the expression of Bcl-2 and reduce the expression of Bax, Caspase-3, and Caspase-9, and EGCG supplementation could mitigate estrogen deficiency associated bladder dysfunction in a dose-dependent manner through its antioxidant, anti-fibrosis and anti-apoptosis effects^5,6^.

However, in this study, the conditions of MetS combined with ovarian hormone deficiency seemed to be more complicated than ovarian hormone deficiency alone. It should be emphasized that EGCG was administrated at the same time as HFHS diet in the present study. Our findings suggested that EGCG pretreatment could prevent MetS and ovarian hormone deficiency related oxidative stress through mitochondria and ER-mediated apoptotic pathway. Thus, decreasing oxidative stress as well as enhancing mitochondrial function might be important treatment strategies for the status of MetS combined with ovarian hormone deficiency^8^.

The limitation in statistical analysis would need to be addressed. Strengthened multiple test was not performed in this study. Several important features were involved in the cellular mechanism of bladder overactivity. The numbers of variables were varied in every aspect of each table/figure. The corrected criteria of p values would be different in every table and figure. In order not to confuse our readers, we present basic statistical results. However, the conclusions were not affected, because there were no more than five variables or genes in each analysis, and the key data of our major conclusion were with p < 0.01 significance.

## Conclusions

MetS and ovarian hormone deficiency could enhance the generation of oxidative stress mediated by mitochondria and ER apoptosis pathways, leading to significant bladder apoptosis and interstitial fibrosis. Oxidative stress, apoptosis and mitochondrial dysfunction might be important factors underlying bladder overactivity and inflammatory process. EGCG administration alleviated bladder overactivity induced by MetS and ovarian hormone deficiency, attenuated oxidative stress and apoptosis of bladder, and diminished the over-expressions of mitochondrial and ER apoptotic signals.

## Electronic supplementary material

Supplementary Information
