# Supplementary material for: Epigallocatechin-3-gallate alleviates bladder overactivity in a rat model with metabolic syndrome and ovarian hormone deficiency through mitochondria apoptosis pathways
Source: Sci Rep. 2018 Mar 29;8:5358. doi: 10.1038/s41598-018-23800-w (PMC5876359; doi:10.1038/s41598-018-23800-w)
Supplement: Supplementary file 1 — Supplementary Information [file 41598_2018_23800_MOESM1_ESM.pdf]

Epigallocatechin-3-gallate alleviates bladder overactivity in a rat model with metabolic syndrome and ovarian hormone depletion through mitochondria apoptosis pathways

Yi-Lun Lee[1,2], Kun-Ling Lin[3,4], Bin-Nan Wu[1,5], Shu-Mien Chuang[6], Wen-Jeng Wu[1,7,8,9], Yung-Chin Lee[7,8,10],Wan-Ting Ho[7,8], Yung-Shun Juan[1,7,8,9]

[1]Graduate Institute of Medicine, College of Medicine, Kaohsiung Medical University, Kaohsiung, Taiwan;
[2]Department of Urology, Sinying Hospital, Ministry of Health and Welfare, Tainan, Taiwan;
[3]Graduate Institute of Clinical Medicine, College of Medicine, Kaohsiung Medical University, Kaohsiung, Taiwan;
[4]Department of Obstetrics and Gynecology, Kaohsiung Medical University Hospital, Kaohsiung, Taiwan;
[5]Department of Pharmacology, College of Medicine, Kaohsiung Medical University, Kaohsiung, Taiwan;
[6]Translational Research Center, Cancer Center, Department of Medical Research, Kaohsiung Medical University, Kaohsiung, Taiwan;
[7]Department of Urology, College of Medicine, Kaohsiung Medical University, Kaohsiung, Taiwan;
[8]Department of Urology, Kaohsiung Medical University Hospital, Kaohsiung, Taiwan;
[9] Department of Urology, Kaohsiung Municipal Ta-Tung Hospital, Kaohsiung, Taiwan;
[10]Department of Urology, Kaohsiung Municipal Hsiao-Kang Hospital, Kaohsiung, Taiwan

Correspondence:
Yung-Shun Juan MD, PhD;
Department of Urology, College of Medicine, Kaohsiung Medical University, Kaohsiung, Taiwan
Address: 100 Shih-Chuan 1st Road Sanmin District, Kaohsiung City 807, Taiwan
Telephone number: 886-7-3121101
Fax number: 886-7-3506269
E-mail address: juanuro@gmail.com;
Telephone number: 886-7-3506269

Keywords: EGCG, bladder overactivity, metabolic syndrome, ovarian hormone, apoptosis

Supplementary Information:

1. Cropped Western blots in different groups and different animals

Western full blot

Protein :	ATPB
Catalog number: <Abcam> ab14730	
Molecular weight : 52 kDa	
Recommend 1*Ab ratio : 1:2000	
2*Ab : Mouse IgG1	

1. Control 2. MetS 3. MetS+OVX 4. MetS+OVX+EGCG 5. MetS+EGCG

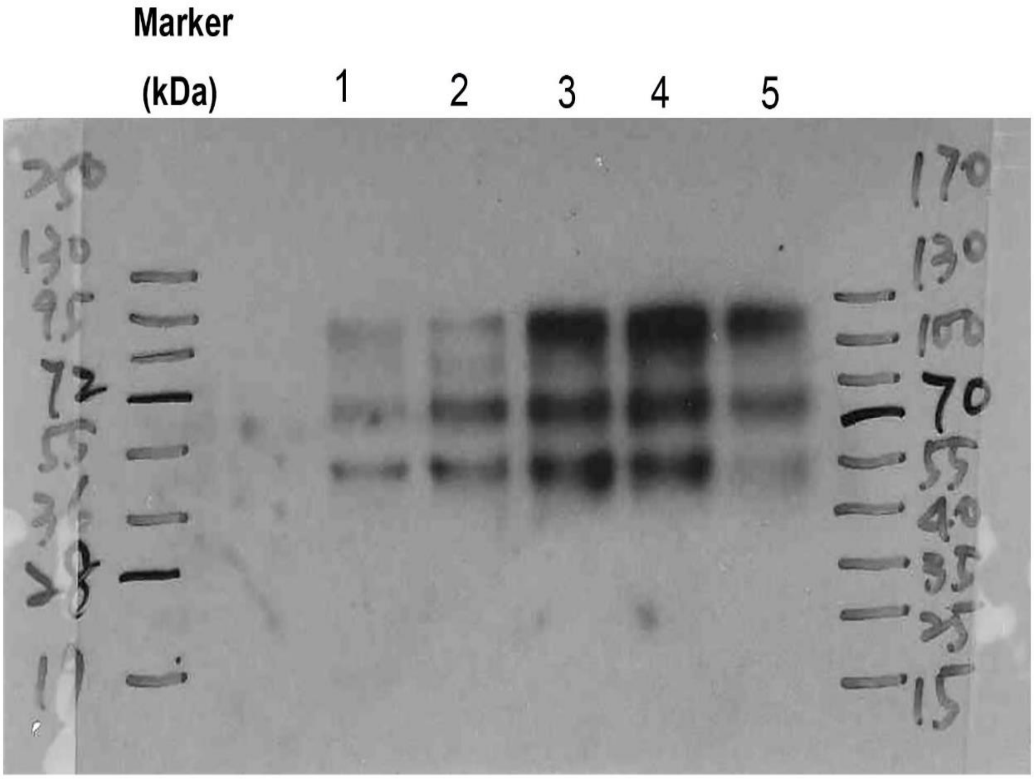

Western full blot

Protein : COX2
Catalog number: <Cayman> Item NO.160112
Molecular weight : 72 kDa
Recommend 1*Ab ratio : 1:1000
2*Ab : Mouse IgG1

1. Control 2. MetS 3. MetS+OVX 4. MetS+OVX+EGCG 5. MetS+EGCG

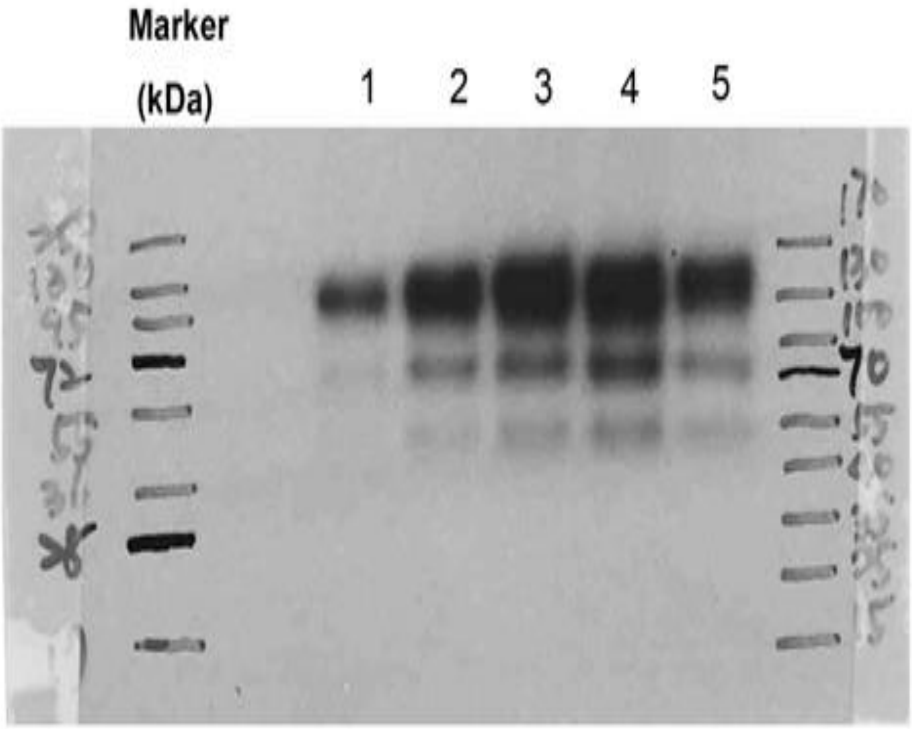

Western full blot

Protein : SDHA
Catalog number: <Abcam> ab14715
Molecular weight : 70 kDa
Recommend 1*Ab ratio : 1:1000
2*Ab : Mouse IgG1

1. Control 2. MetS 3. MetS+OVX 4. MetS+OVX+EGCG 5. MetS+EGCG

Marker

(kDa) 1 2 3 4 5

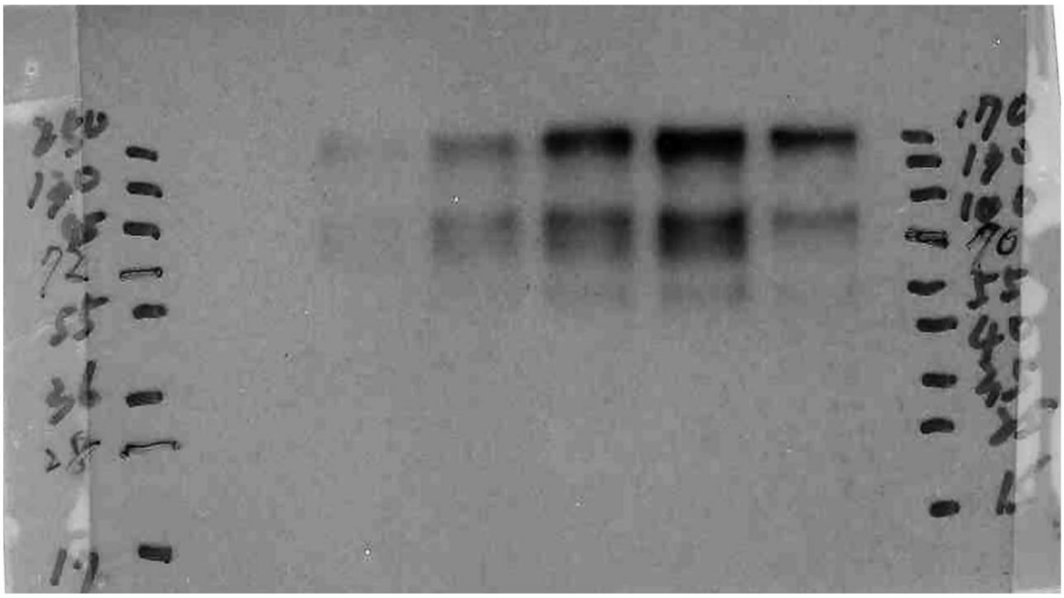

Western full blot

Protein : b-actin
Catalog number: <   >
Molecular weight : 43 kDa
Recommend 1*Ab ratio : 1:10000
2*Ab : Mouse IgG

1. Control 2. MetS 3. MetS+OVX 4. MetS+OVX+EGCG 5. MetS+I

Marker (kDa) 1 2 3 4 5

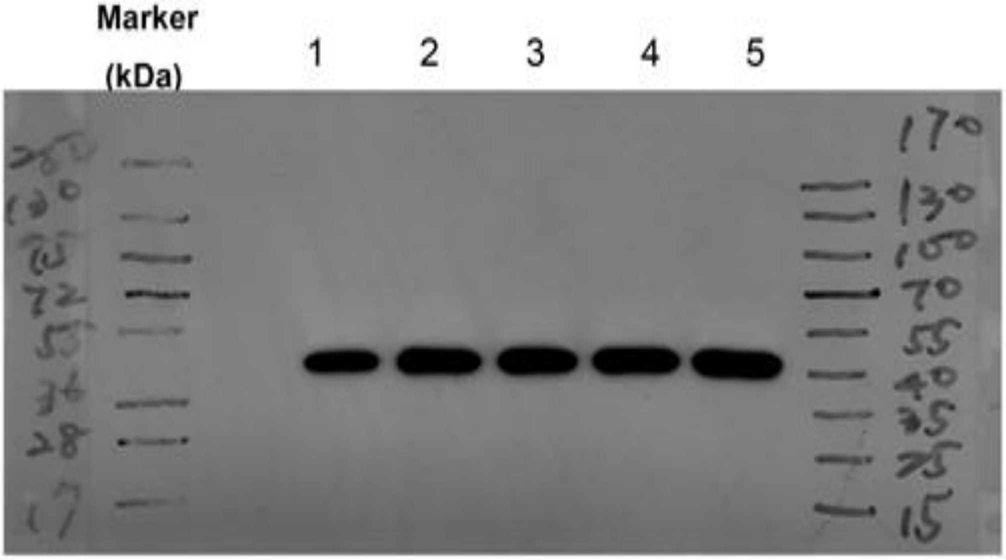

Marker (kDa) 1 2 3 4 5

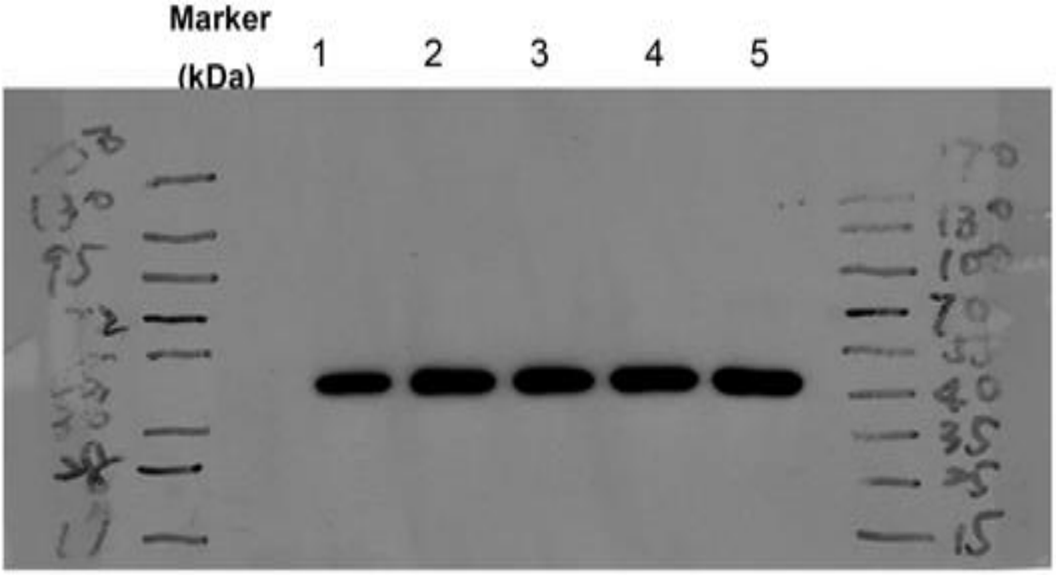

Western full blot

Protein :	Fibronectin
Catalog number: <Millipore> MAB1926	
Molecular weight : ~250 kDa	
Recommend 1*Ab ratio : 1:1000	
2*Ab : Mouse IgG	

1. Control 2. MetS 3. MetS+OVX 4. MetS+OVX+EGCG 5. MetS+EGCG

Marker

(kDa) 1 2 3 4 5

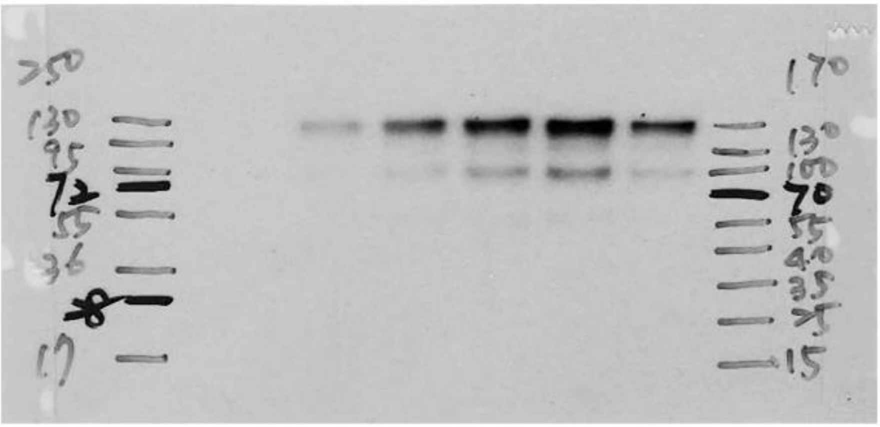

Marker

(kDa) 1 2 3 4 5

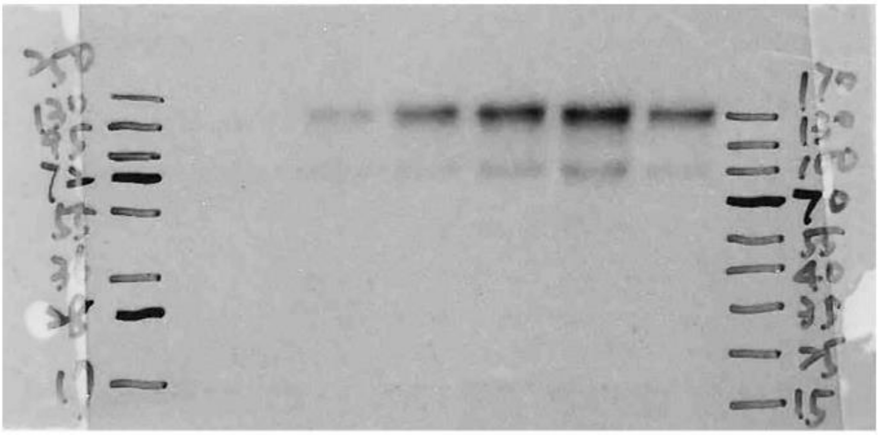

Protein : Nitrotyrosine
Catalog number: <ALEXIS> NOY-7A5
Molecular weight :   kDa
Recommend 1*Ab ratio : 1: 1000
2*Ab : Mouse IgG

1. Control 2. MetS 3. MetS+OVX 4. MetS+OVX+EGCG 5. MetS+EGCG

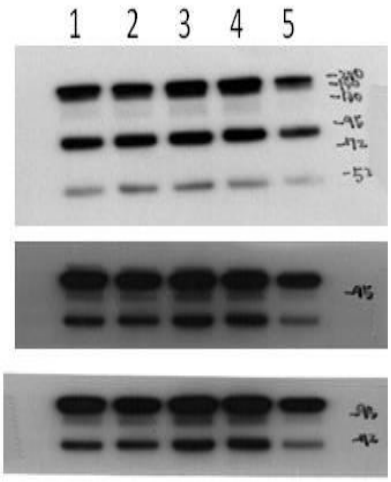

Protein : COX2
Catalog number: <Cayman> Item NO.160112
Molecular weight : 72 kDa
Recommend 1*Ab ratio : 1:1000
2*Ab : Mouse IgG1

1. Control 2. MetS 3. MetS+OVX 4. MetS+OVX+EGCG 5. MetS+EGCG

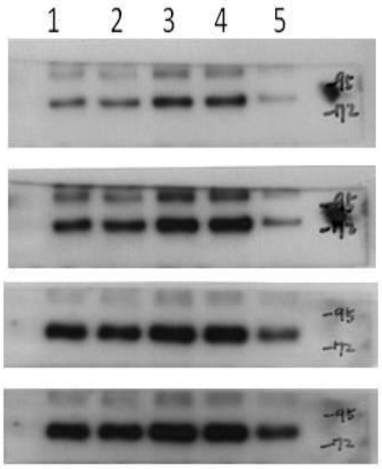

Protein : BAX
Catalog number: <Proteintech > 50599-2-Ig
Molecular weight : 21~24 kDa
Recommend 1*Ab ratio : 1/1000
2*Ab : Rabbit IgG

1. Control 2. MetS 3. MetS+OVX 4. MetS+OVX+EGCG 5. MetS+EGCG

1 2 3 4 5 1 2 3 4 5

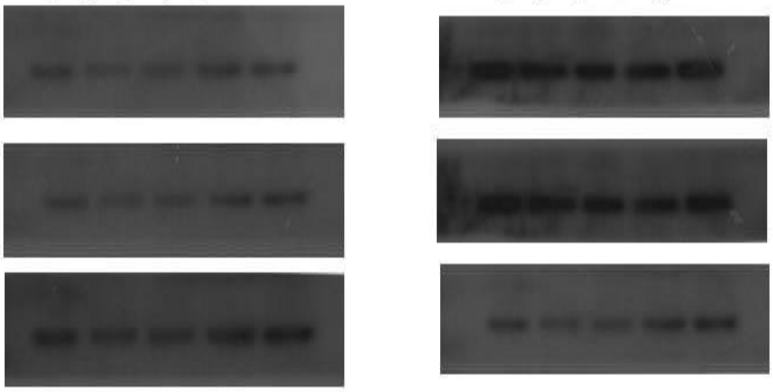

Protein : b-actin
Catalog number: < >
Molecular weight : 43 kDa
Recommend 1*Ab ratio : 1:10000
2*Ab : Mouse IgG

1. Control 2. MetS 3. MetS+OVX 4. MetS+OVX+EGCG 5. MetS+EGCG

1 2 3 4 5

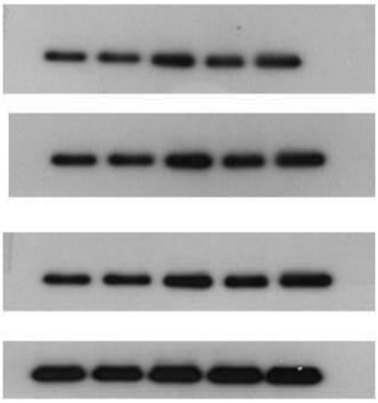

Protein : GAPDH
Catalog number: <Millipore > MAB374
Molecular weight : 36 kDa
Recommend 1*Ab ratio : 1/10000
2*Ab : Mouse IgG1

1. Control 2. MetS 3. MetS+OVX 4. MetS+OVX+EGCG 5. MetS+EGCG

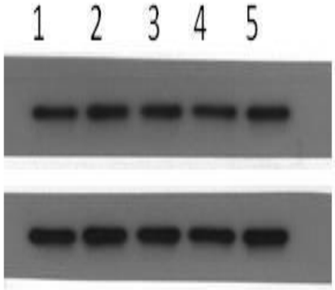

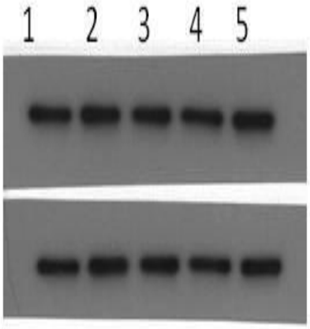

Supplementary Figures

(Supplementary Figure 1)

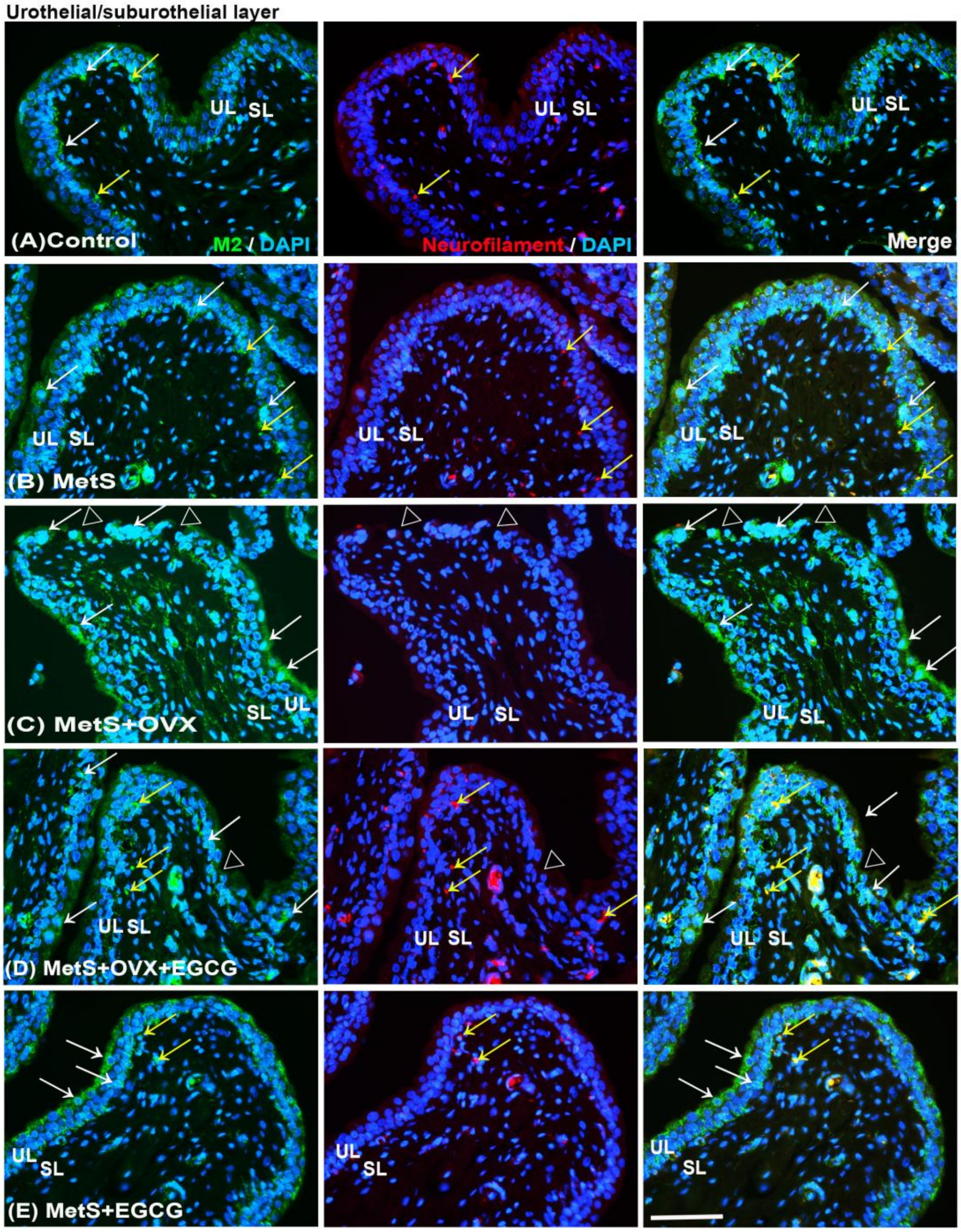

Supplementary Figure S1. Double immunostaining analysis of M2 receptor (red, left panels) and neurofilament (green, middle panels) protein for bladder was shown in the Control group (A), the MetS group (B), the MetS+OVX group (C), the MetS+OVX+EGCG group (D) and the MetS+EGCG group (E). The merged image from left and middle panels (yellow, right panels) was shown. However, the M2 expression (green) co-stained with neurofilament (red) was mainly distributed in the basal layer of the urothelium (yellow arrows). Nuclear DNA was labeled with DAPI

(blue). Scare bar=100 μm.

(SupplementaryFigure 2)

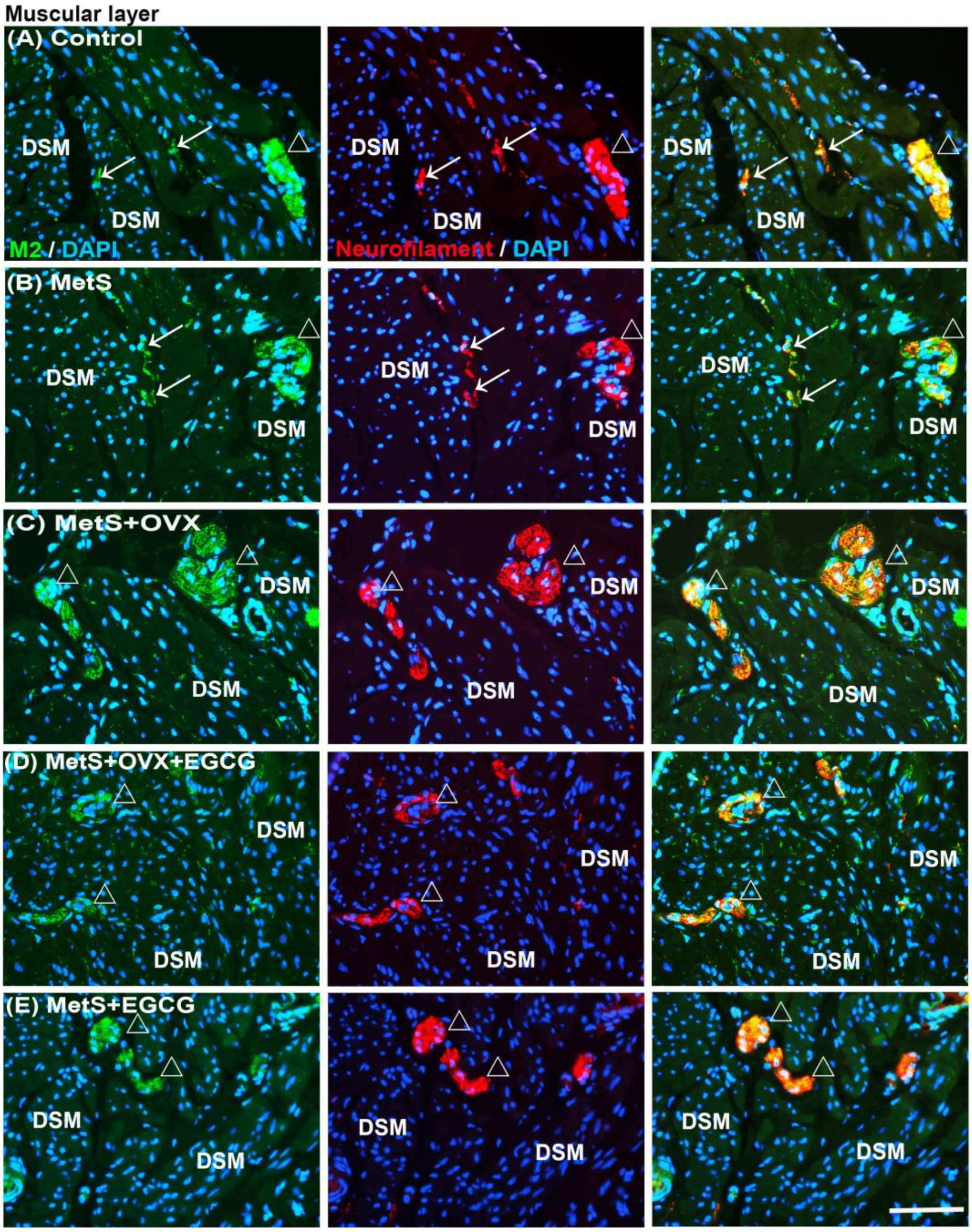

Supplementary Figure S2. Co-labeling of M2 (red, left panels) and neurofilament (green, middle panels) (arrows) for bladder was shown in the detrusor smooth muscle (DSM) layer. Five groups included the Control group (A), the Met group (B), the MetS+OVX group (C), the MetS+OVX+EGCG group (D) and the MetS+ EGCG group (E). M2 and neurofilament proteins were strongly co-labeled in the neural gangion (arrowheads) between DSM bundles in the Control and the MetS groups. Scare bar=100 μm.
